# General anxiety, dental anxiety, digit sucking, caries and oral hygiene status of children resident in a semi-urban population in Nigeria

**DOI:** 10.1186/s12903-018-0529-z

**Published:** 2018-04-20

**Authors:** Morenike O. Folayan, Kikelomo A. Kolawole, Nneka K. Onyejaka, Hakeem O. Agbaje, Nneka M. Chukwumah, Titus A. Oyedele

**Affiliations:** 1 Department of Child Dental Health, Obafemi Awolowo University, Ile-Ife, Nigeria; 2 Oral Habit Study Group, Ile-Ife, Nigeria; 3 Department of Child Dental Health, Obafemi Awolowo University Teaching Hospitals Complex, Ile-Ife, Nigeria

**Keywords:** Anxiety, General, Dental, Caries, Oral hygiene, Children, Nigeria

## Abstract

**Background:**

Digit sucking can represent untreated anxiety or other emotional problems. The aim of this study was to determine if digit sucking is a predictor of general anxiety and dental anxiety; and if general and dental anxiety are associated with caries and oral hygiene status of children resident in sub-urban Nigeria.

**Methods:**

This was a secondary data analysis of a household survey conducted in Ile-Ife, Nigeria. The level of general anxiety and dental anxiety of 450 6 to12 year old children were measured using the Revised Child Manifest Anxiety Scale and Dental Subscale of the Child Fear Survey Schedule respectively. Presence of digit sucking habit, caries and oral hygiene status were determined. General anxiety and dental anxiety scores were dichotomized into low and high levels respectively. Logistic regression was conducted to determine if digit sucking was a predictor of general anxiety and dental anxiety; and if general anxiety and dental anxiety were predictors caries and good oral hygiene status. Adjustments were made for age and sex.

**Results:**

Digit sucking is not a significant predictor of dental anxiety (*p* = 0.99) and general anxiety (*p* = 0.79). Children with high general anxiety (AOR: 5.02; 95% CI: 2.9–9.74; *p* <  0.001) and high dental anxiety (AOR: 1.74; 95% CI: 1.15–2.65; *p* = 0.009) had higher odds of having caries and good oral hygiene respectively.

**Conclusion:**

Digit sucking was not a significant predictor of general anxiety and dental anxiety. General and dental anxiety however, had effects on the likelihood of having caries and good oral hygiene.

**Electronic supplementary material:**

The online version of this article (10.1186/s12903-018-0529-z) contains supplementary material, which is available to authorized users.

## Background

Non-nutritive sucking (NNS) habits are common oral habits, observed in children and in some adults. Digit sucking and nail biting are referred to as nervous NNS habits [1]. These habits are prevalent in normally developing preschool children, and they reflect the state of the mood [1]. Some suggested etiological factors for nail biting include anxiety, stress, loneliness, imitation of others, heredity and inactivity [2]. Nail biting is considered a transitional behavior from thumb sucking [2].

Digit sucking is a normal behavior for young children because they are born with a natural sucking instinct [3]. For most infants, this instinct can last up to the sixth month of life, while for some sucking the habit can continue beyond the sixth month of life when it becomes a soothing and comforting behavior for scared, hungry, sleepy, bored or anxious children [3]. When the habit persists beyond 4 years of age, it can represent untreated anxiety or other emotional problems [4].

General anxiety has been well linked to dental anxiety. Winer [5] suggested that dental fear in children might not be a specific form of fear, but instead may reflect general fear, as there is usually a decline in the prevalence of both general and dental fear with age: the same kind of decline observed with thumb sucking. Klingberg [6] therefore, suggested that children predisposed to general fears should be regarded as having a potential risk of developing dental fear. However, Neverlien [7] found no direct associations between general fear and self-reported dental anxiety, though there was a significant correlation between these two factors for girls who showed signs of clinical anxiety. Folayan et al. [8] however, showed a significant but moderate correlation between dental anxiety and general anxiety.

While Tyron [9] concluded there was no relationship between digit sucking and general anxiety [9], Mahalski and Stanton [10] demonstrated such a relationship through a 5 year longitudinal study. The link between digit sucking and dental anxiety has however not been demonstrated. There is a possibility for such link since prior studies had shown an association between general anxiety and dental anxiety [5, 6, 8]; and an association between digit sucking, other NNS habits and general anxiety [10]. This study will therefore determine if digit sucking is a predictor of dental and general anxiety in children.

Digit sucking has deleterious effects on the oral health of children older than 4 years [11, 12]. Shimura et al. [13] had highlighted the role of emotional stress and the associated psychosomatic responses as a predisposing factor for caries. The association between dental anxiety and poor oral hygiene in adults has also been highlighted [14–16]. We authors found no information in the dental literature on general and dental anxiety being predictors of caries and oral hygiene status in children. Though de Carvalho et al. [17] demonstrated a correlation between dental anxiety oral hygiene frequency and caries, their target population were adolescents not children. This study will therefore, also determine if general anxiety and dental anxiety are associated with caries and oral hygiene status of children resident in sub-urban Nigeria.

## Method

This study retrieved the data of children 6 to 12 years old from the data of a larger study conducted to explore the relationship between non-nutritive oral habits and caries [18]. The primary study was a cross sectional study utilising a household survey for study participants’ recruitment. A household survey was conducted in order to recruit a representative sample of children from the community since 40.0% of primary school aged children and 60.0% of secondary school aged children are out of school [19].

### Study setting

The study was conducted in Ife Central Local Government area of Osun State, a semi-urban area. Ife Central was chosen as the study location due to its proximity to the Obafemi Awolowo University and the Obafemi Awolowo University Teaching Hospitals Complex, the host institutions of the authors.

Participants were recruited from the National Population Enumeration sites in the Local Government Area. The Enumeration sites were the same used for the 2010 National Antenatal Sero-sentinel Survey [20] and the 2012 National Adolescent Reproductive Health Survey [21]. These study sites were selected because it was assumed that participants in these geographical sites may have been familiar with the conduct of such surveys and thus, be more open to discussing with the field workers.

### Study population

The study population for the primary study included 1–7 year old children whose parents gave consent for study participation, and 8–12 year old children who gave assent for study participation in addition to parental consent. Only children who were living with their biological parents or legal guardians and who were at home at the time of data collection were included in the study. The lower age limit for study participants was fixed at 6 years because some of the study tools were designed to collect data for children 6 years and above. The children included in the study were therefore those with in the age-range of children with mixed dentition.

### Sample size

Sample size for the primary study as calculated using Leslie Fischer’s formula [22] for study population > 10,000. A previous study [23] reported a prevalence of 34.1% for oral habits in 4–15 year old Nigerian children. Based on a prevalence of 34.1%, it was found that it would be necessary to examine 1011 children to capture 345 children with oral habits, with a fall out rate of 10%.

### Sampling technique

The sampling procedure used a multi-stage cluster sampling method to select eligible persons. First, there was the random selection of enumeration areas within the Local Government Area. Next, every third household on each street at the enumeration areas was identified for study participant recruitment. In each household, eligible individuals were listed and one eligible child randomly selected for study participation using balloting.

### Study procedure

Experienced trained field workers administered a structured questionnaire developed in English to collect data for the study using an approach had had been used successfully for prior studies conducted in multi-lingual communities in Nigeria [24–29].

The field workers collected information from the respondent and submitted the completed questionnaires to the survey supervisor daily. The supervisor reviewed all filled questionnaires and raised queries where gaps were identified in the filled questionnaire, or the consenting process. The queries were addressed latest by the next day by the field worker where this was feasible. This may involve returning to the household to collect missing data or the need to entire essential documentation details in the filled questionnaires.

The questionnaires assessing dental anxiety and general anxiety were only administered to children 6–12 years old. The questionnaires were filled by the mothers of children 6–7 years and by children 8–12 years. Mothers were requested to fill the questionnaire on behalf of their children because prior studies conducted in the same environment showed that the correlation between child’s general and dental anxiety level best correlated with the mother’s assessment of these situations when compared to correlation with the father’s assessment [8]. Dental and general anxiety levels were classified as high or low using the cut off established for the study population by Folayan et al. [8]. However, where the mother was unavailable, fathers completed the questionnaires.

### Data collection tools

The questionnaire asked for details on the child’s socio-demographic characteristics (age, sex), if digit sucking habit was present, general anxiety and dental anxiety (Sections 1, 3, 18 and 19 of the Additional file 1 respectively). Details of the child’s medical and dental history which explored possible medical and dental health issues that could interfere with oral health were also collected (Section 17 of the Additional file 1). Any child who had any form of cognitive impairment was excluded from the study.

#### General anxiety

The Revised Child Manifest Anxiety Scale (RCMAS) [30] was used to measure level and nature of self-reported trait anxiety. The instrument had been used on prior studies conducted among children in Nigeria [31, 32] and its reliability for use among school children in Nigeria determined [19]. Cross-cultural validity of the tool had also been determined [33]. It consists of 28 anxiety items and 9 lie (social desirability) yes-or-no items. A response of “Yes” indicates that the item is descriptive of the subject’s feelings or actions, whereas a response of “No” indicates that the item is generally not descriptive. Scores are provided for total anxiety and four sub-scales namely the 10 item physiological anxiety scale, the 11 item worry/oversensitivity scale, the 7 item social concerns/concentration scale, and the 9 item lie scale. The possible total score ranged from 0 to 37. Scores were derived from affirmative responses. A high score indicates a high level of anxiety or lie [23]. A prior study had used a cut-off point of 19 to identify children experiencing clinically significant levels of anxiety [33]. For this study, children with scores 19 and below were categorized as having low general anxiety and those with scores above 19 were categorized as having high trait anxiety.

#### Dental anxiety

The dental anxiety of each child was assessed using Dental Subscale of the Child Fear Survey Schedule (CFSS-DS) described by Cuthbert and Melamed [34]. The CFSS-DS is a 5-point Likert scale with scores ranging from 1 (not afraid at all) to 5 (very afraid) for each of the 15 items. These covered different aspects of the dental situation. Total scores ranged from 15 to 75. The scale had been used in a prior study conducted in the same population in Nigeria, to measure dental anxiety [23]. Children with scores equal to and less than median score for the group were classified as having low anxiety while those who scored above the median score were classified as having high anxiety. This method of categorization had been used by Folayan et al. [35]. For this study, children with scores below 35 were categorized as having low dental anxiety and those with scores 35 and above were categorized as having high dental anxiety.

### Intra-oral examination

All children eligible to participate in the study had an oral examination conducted in their homes on the day of study visits. The children were examined under natural light while sitting down on the chair, using sterile dental mirrors by trained dentists attached to each field worker. The teeth were examined wet. Intraoral examination was conducted to determine presence of caries and its severity and oral hygiene status. Radiographs were not used in the study.

#### Oral hygiene status

The most commonly used index to assess the oral hygiene status, the oral hygiene index was used for assessment in this study. The Oral Hygiene Index (Simplified) OHI-S described by Greene and Vermillion [36] was used to determine the oral hygiene status. It is composed of the Debris Index and Calculus index, each of which was obtained based on 6 numerical determinations representing the amount of debris or calculus found on the surfaces of the index teeth. 11, 16, 26, 31, 36 and 46 and 51, 55, 65, 71, 75, 85 in the permanent and deciduous dentitions respectively. For each individual, the debris and calculus scores were totaled and divided by the number of surfaces scored. The scores were graded as 0.0–1.2 = Good oral hygiene, 1.3–3.0 = Fair oral hygiene and > 3.1 = Poor oral hygiene.

#### Caries profile

The teeth were examined for caries after the OHI-S was determined. Debris was removed from the wet teeth using gauze prior to assessment for caries status. The teeth present were charted using the FDI tooth numbering system. Caries diagnosis was based on the recommendation of the World Health Organisation Oral Health Survey methods [37]. The caries status was assessed using the Decayed Missing and Filled/decayed missing and filled teeth (DMFT/dmft) index. For ease of analysis, caries status was further divided into caries present or caries absent. Children were classified as having caries present when a tooth was identified to be decayed.

To arrive at a dmft/DMFT score for an individual child, three values were determined: the number of teeth with carious lesions, the number of extracted teeth due to caries, and the number of teeth with fillings or crowns [38]. Parents of children were asked to explain the loss of any teeth that was not found during the oral examination. Only tooth extracted due to caries were recorded as missing. The number of teeth are summed together to give the dmft score for the primary dentition and the DMFT score for the permanent dentition.

#### Calibration of examiners

Clinical investigators were postgraduate Paedodontists and Orthodontists residents. They were calibrated on the use of the WHO criteria for caries diagnosis and the OHI-S. The intra-examiner scores ranged from 0.89 to 0.94, while inter-examiner variability ranged from 0.82 to 0.90 for caries detection and OHI-S [18].

### Data analysis

Descriptive analysis was conducted using a variety of measures of location and dispersion. This was represented as Tables. A test of association was conducted to determine the association between the general anxiety subscales and the presence of caries, or the oral hygiene status. Multivariate logistic regression was conducted to determine the predictors of presence of caries and good oral hygiene; and if digit sucking was a predictor of general anxiety and dental anxiety. The age of the study participants were grouped into two: 6–8 years and 9–12 years. The effect of age and sex were controlled for. Statistical analysis was conducted with Intercooled STATA (release 12) for windows. Statistical significance was inferred at *p* ≤ 0.05.

## Results

Four hundred and ninety seven participants were eligible to participate in the study. Only 450 (90.5%) participants had data complete enough for analysis of information on both dental and general (state and trait) anxiety. These included 226 (50.2%) 6-8 year olds and 222 (49.3%) male participants. Very few study participants (4.5%) had poor oral hygiene and very few sucked their digits (6.0%). Table 1 highlights the profile of the study participants.

**Table 1:** Frequency distribution of demographic variables, caries status, oral hygiene status and anxiety status (*N* = 450)

Demographic profile	*N* = 450 Number (%)
Age	
6 years – 8 years	226 (50.2%)
9 years – 12 years	224 (49.8%)
Gender	
Male	222 (49.3%)
Female	228 (50.7%)
Caries status	
Caries present	76 (16.6%)
Caries free	374 (83.1%)
dmft	
0	386 (85.8%)
1–2	48 (10.7%)
3–6	16 (3.5%)
DMFT	
0	428 (95.1%)
1–2	18 (4.0%)
3–4	4 (0.9%)
Oral hygiene status	
Good	172 (38.4%)
Fair	256 (57.1%)
Poor	20 (4.5%)
Dental anxiety	
Low	226 (50.2%)
High	224 (49.8%)
General anxiety	
Low	392 (87.1%)
High	58 (12.9%)
Digit sucking	
Present	27 (6.0%)
Absent	423 (94.0%)

The general anxiety scores measured using the RCMAS ranged from 0 to 36. The mean score was 11.8 ± (7.6) and the median score was 9. The mean physiological anxiety score was 2.42 ± (2.36). The mean worry/oversensitivity score was 3.03 ± (3.34). The social concerns/concentration score was 1.77 ± (2.01). The mean lie score was 4.52 ± (1.84). Three hundred and ninety two (87.1%) participants had low clinically significant trait anxiety (anxiety scores less than 19) while 58 (12.9%) participants had high clinically significant trait anxiety (anxiety scores between 19 and 28).

The dental anxiety scores measured using the CFSS-DS ranged from 15 to 75. The mean score was 38.6± (14.4) and the median score was 35. Two hundred and thirteen (47.3%) participants had low dental anxiety (dental anxiety scores less than 35) while 237 (52.7%) respondents had high dental anxiety (dental anxiety scores 35 and above).

Seventy six (16.6%) participants had caries. The dmft scores ranged from 0 to 6 and the DMFT scores ranged from 0to 4. The mean dmft was 0.29 ± (0.84) and the mean DMFT was 0.08 ± (0.44).

### Digit sucking and anxiety

Table 2 highlights the association between digit sucking, dental anxiety and general anxiety having controlled for age and sex. Digit sucking was not significantly associated with dental anxiety (*p* = 0.99) and general anxiety (*p* = 0.79). Neither was it a general anxiety (AOR: 0.83; 95% CI: 0.23–3.07) or dental anxiety (AOR: 1.01; 95% CI: 0.44–2.31) a predictor of digit sucking habit.

**Table 2:** Frequency distribution and logistic regression analysis of digit sucking as predictor of general anxiety and dental anxiety (*N* = 450)

Variables	Digit sucking				Simple regression		Multiple regression	
	Absent (*N* = 423)	Percent	Present (*N* = 27)	Percent	OR (95% CI)	*p*-value	AOR (95% CI)	*p*-value
Sex								
Male	206	48.7	16	59.3	1	–	1	–
Female	217	51.3	11	40.7	0.65 (0.30–1.44)	0.29	0.66 (0.29–1.46)	0.30
Age group								
6–8 years	212	50.1	14	51.9	1	–	1	–
9–12 years	211	49.9	13	48.1	0.93 (0.43–2.03)	0.86	0.96 (0.43–2.11)	0.91
Dental Anxiety								
Low	212	50.1	14	51.9	1	–	1	–
High	211	49.9	13	48.1	0.93 (0.43–2.03)	0.86	1.01 (0.44–2.31)	0.99
General anxiety								
Low	368	87.0	24	88.9	1	–	1	–
High	55	13.0	3	11.1	0.84 (0.24–2.87)	0.78	0.83 (0.23–3.07)	0.79

### Caries and anxiety

Table 3 shows the results of the test of association between the general anxiety subscale and caries status. Children who had caries had significant higher means scores (*p* <  0.001) on the physiological anxiety, worry/oversensitivity and social concerns/concentration scales respectively.

**Table 3:** Association between general anxiety subscales and caries status

Subscales	Caries status	Number	Mean ± sd	t	df	*p* value
Physiological anxiety	Present	76	3.4 ± 2.9	3.9	448	< 0.001
	Absent	374	2.2 ± 2.2			
Worry/oversensitivity	Present	76	4.6 ± 4.1	4.5	448	< 0.001
	Absent	374	2.7 ± 3.1			
Social concerns/concentration	Present	76	2.5 ± 2.4	3.6	448	< 0.001
	Absent	374	1.6 ± 1.9			
Lie	Present	76	4.6 ± 1.9	0.4	448	0.72
	Absent	374	4.5 ± 1.8			

Table 4 highlights the predictors of presence of caries. Children who had high general anxiety (OR: 5.07; 95% CI: 2.79–9.20; *p* <  0.001) had higher odds of having caries when compared with children with low general anxiety. Also children who had high dental anxiety (OR: 1.69; 95% CI: 1.02–2.80; *p* = 0.04) had higher odds of having caries when compared with children with low dental anxiety. After adjusting for age, sex and dental anxiety, general anxiety was still a significant predictor of presence caries: children who had high general anxiety (AOR: 5.02; 95% CI: 2.59–9.74; *p* <  0.001) had higher odds of having caries when compared with children with low general anxiety.

**Table 4:** Frequency distribution and logistic regression on predictors of presence of caries (*N* = 450)

Variables	Caries				Simple regression		Multiple regression	
	Absent (*N* = 374)	Percent	Present (*N* = 76)	Percent	OR (95% CI)	p-value	AOR (95% CI)	p-value
Sex								
Male	189	50.5	33	43.4	1	–	1	–
Female	185	49.5	43	56.6	1.33 (0.81–2.19)	0.26	1.35 (0.80–2.27)	0.26
Age group								
6–8 years	185	49.5	41	53.9	1	–	1	–
9–12 years	189	50.5	35	46.1	0.84 (0.51–1.37)	0.48	0.91 (0.54–1.54)	0.73
Dental Anxiety								
Low	196	52.4	30	39.5	1	–	1	–
High	178	47.6	46	60.5	1.69 (1.02–2.80)	0.04	1.00 (0.56–1.79)	0.99
General anxiety								
Low	341	91.2	51	67.1	1	–	1	–
High	33	8.8	25	32.9	5.07 (2.79–9.20)	< 0.001	5.02 (2.59–9.74)	< 0.001

### Oral hygiene and anxiety

Table 5 shows the results of the test of association between the general anxiety subscale and oral hygiene status. Children with fair oral hygiene had significant lower mean scores on each of the subscales.

**Table 5:** Association between general anxiety subscales and oral hygiene status

Subscales	Oral hygiene status	Number	Mean ± sd	F(df)	p value
Physiological anxiety	Good	172	2.9 ± 2.6	5.67 (2, 445, 447)	0.004
	Fair	256	2.1 ± 2.1		
	Poor	20	2.7 ± 2.3		
Worry/oversensitivity	Good	172	3.7 ± 3.7	7.52 (2, 445, 447)	0.001
	Fair	256	2.5 ± 3.0		
	Poor	20	3.9 ± 3.5		
Social concerns/concentration	Good	172	2.1 ± 2.3	5.39 (2, 445, 447)	0.005
	Fair	256	1.5 ± 1.7		
	Poor	20	2.4 ± 2.4		
Lie	Good	172	4.8 ± 1.8	3.55 (2, 445, 447)	0.03
	Fair	256	4.3 ± 1.8		
	Poor	20	4.7 ± 1.9		

Table 6 highlights the predictors of good oral hygiene. Children who had high dental anxiety (OR: 2.27; 95% CI: 1.52–3.32; p <  0.001) had higher odds of having good oral hygiene when compared with children with low dental anxiety. Also children who had high general anxiety (OR: 2.38; 95% CI: 1.35–4.20; *p* = 0.002) had higher odds of having good oral hygiene when compared with children with low general anxiety. After adjusting for age, sex and general anxiety, dental anxiety was still a significant predictor of presence good oral hygiene: children who had high dental anxiety (AOR: 1.87; 95% CI: 1.23–2.84; *p* = 0.003) had higher odds of having good oral hygiene when compared with children with low dental anxiety. Age was also a significant predictor of good oral hygiene children in the unadjusted and adjusted models: older children had lower odds of having good oral hygiene when compared with younger children (AOR: 0.66; 95% CI:0.44–0.98; *p* = 0.04).

**Table 6:** Frequency distribution and logistic regression analysis on the predictors of good oral hygiene (*N* = 448)

Variables	Oral Hygiene				Simple regression		Multiple regression	
	Poor (*N* = 276)	Percent	Good (*N* = 172)	Percent	OR (95% CI)	*p*-value	AOR (95% CI)	*p*-value
Sex								
Male	142	51.4	78	45.3	1	–	1	–
Female	134	48.6	94	54.7	1.28 (0.87–1.87)	0.21	1.25 (0.84–1.86)	0.27
Age group								
6–8 years	126	45.7	100	58.1	1	–	1	–
9–12 years	150	54.3	72	41.9	0.61 (0.41–0.89)	0.01	0.66 (0.44–0.98)	0.04
Dental Anxiety								
Low	160	58.0	65	37.8	1		1	–
High	116	42.0	105	61.2	2.27 (1.52–3.38)	< 0.001	1.87 (1.23–2.84)	0.003
General anxiety								
Low	251	90.9	139	80.8	1	–	1	–
High	25	9.1	33	19.2	2.38 (1.35–4.20)	0.002	1.71 (0.94–3.11)	0.08

## Discussion

The study highlighted the association between dental anxiety, general anxiety, digit sucking, caries and oral hygiene status of children in the age range for mixed dentition, in the study population. We found that digit sucking was not a significant predictor of dental anxiety or general anxiety. The prevalence of high dental anxiety was high in the study population; children with high dental anxiety and younger children were significantly more likely to have good oral hygiene. About an eight of the population had high general anxiety; children with high general anxiety were significantly more likely to have caries.

First, like Tyron [9] and unlike Mahalski and Stanton [10], we found that digit sucking was not a significant predictor of general anxiety and dental anxiety in this study population. We however were unable to explain these observations though we assume it may be linked with the ways culture influences expression of anxiety: we assume that the African culture promotes internalization of problems and its expressions unlike other cultures where externalizing problems and anxiety are welcome and accepted [8].

Second, unlike many prior studies that had found an association between dental anxiety and the increased risk for caries [39–44], our studies could not establish such association. Some of these studies had conducted bivariate analysis (tests of associations) to establish these associations [39, 40] and others had conducted the studies in older children [34, 35]. Studies that have conducted more robust analysis using logistic regression models reported an association between presence of caries and dental anxiety in older children [43, 44]. The difference in study methodology including differences in the age of the study population and method of data analysis, are factors that can significantly influence study outcome. Our study illustrated this in that with simple logistic regression analysis, dental anxiety was associated with presence of caries. However, when the model was adjusted for age, sex and general anxiety, the observed significance was lost. A few other studies [45–47] had also found no association between presence of caries and dental anxiety.

Third, we also noticed that age and sex were not predictors of caries for children with mixed dentition in this study population. Other studies on dental caries in the mixed dentition had reported similar findings [48, 49] while others had reported observations different from ours [40, 50]. This disparity in study findings may point to residential and cultural differences in risk factors for caries. ‘Genderization’ of diseases and disease processes are also a reflection of how societies and communities ‘genderize’ behaviors that increase risk for diseases [51–53]. Ile-Ife is still considered a sub-rural area where the impact of genderized’ behaviors is seen much later in life than during the mixed dentition stage. Thus, children and teenagers still, for the most part, have homogenized behaviors [54] with distinct age and sexual behaviors occurring at a later age than observed in urbanized communities. Such differences in behavior like being disorganized, self-consciousness and low esteem, increased independency [55] may increase the risk for caries [56]. The homogenized behavior of children in this study population may be a reason why we did not observe significant sexual and age difference in their caries profile.

Fourth, we observed age differences in the oral hygiene profile. A prior study had highlighted differences in the oral hygiene profile of children with primary dentition (1–5 years) and those with mixed dentition (6–12 years) [57]: younger children had better oral hygiene than older children. This study further highlights that for children with mixed dentition, children (6–8 years) had better oral hygiene than teenagers (9-12 years). We feel tooth brushing of children (6-8 years) are still supervised and so increases the chances of having better oral hygiene profile than teenagers who are free from parental supervision of tooth brushing. Our study may be a reflection of this phenomenon. This however, requires further investigation.

Fifth, the independency of the association between general anxiety, dental anxiety, caries and oral hygiene status when adjusted for age and sex, may suggest the independency of the two phenomena – dental anxiety and general anxiety – contrary to the opinion of Winer [5]. Folayan et al. [8] had also reported a moderate but significant correlation between self report of dental anxiety and general anxiety of 8–13 year old children’s in the same study population. This study however, conducted a more robust analysis by adjusting for age and sex as possible confounding variables for dental and general anxiety as highlighted in the literatures. The finding of this regression analysis points to the possibility that the direct relationship observed may be lost in the presence of confounding variables. This postulation needs to be studied further.

This study had a few limitations. First, though the study finding is generalization to the study population, the finding may not be generalizable to a more urban population where culture and behavior of children are more diverse and influenced by multiple variables. Also, this study was based on a secondary data analysis thus the study was not powered to determine differences in digit sucking habit, caries and oral hygiene status based on general anxiety and dental anxiety status. The primary study did not identify caries using radiographs thus it only gave a rough estimate of the prevalence of dental caries in the study population. Examining oral hygiene status in wet conditions without using other aids and without any standardization for the time of examination may also bias the finding. We have however followed standard procedures for assessing oral hygiene status thus making our findings comparable with others that used the OHI-S.Also, when working with children, the lack of attention and poor understanding can generates bias. The finding on the association between general anxiety and caries needs to be taken with caution as the confidence interval is wide. Though we have used a logistic regression analysis to determine digit sucking as a predictor for general and dental anxiety, we recognize that prediction requires a longitudinal design as it involves causality. This study was a cross sectional study thus limited in its ability to truly predict and more powered to determine an association. Despite these limitations, the study has added clarity to our understanding of the association between the variables studied, and suggests that significant associations and effects of general anxiety and dental anxiety on presence of digit sucking, presence of caries and presence of good oral hygiene.

## Conclusion

Digit sucking was not a significant predictor of general anxiety and dental anxiety in the study population. General anxiety significantly increases the likelihood of presence of caries while dental anxiety significantly increases the likelihood of good oral hygiene. Further studies are required to understand how dental anxiety and general anxiety play independent roles as the risk factors for dental caries and oral hygiene when past studies had shown direct relationship between dental anxiety and general anxiety [58, 59] and caries and oral hygiene status [52, 53].

## Additional file

Additional file 1: Individual interview schedule for children aged 0–12 years and their legal guardians. This is the comprehensive questionnaire used for the collection of data for the study. Sections 1, 3, 18 and 19 used to generate the data for this study, are accessible in the questionnaire. (DOC 1091 kb)

## Abbreviations

AOR: Adjusted Odds Ratio
CFSS-DS: Dental Subscale of the Child Fear Survey Schedule
dmft/DMFT: Decay, missing, filled teeth
NNS: Non-nutritive sucking
OR: Odds Ratio
RCMAS: The Revised Child Manifest Anxiety Scale
SD: Standard Deviation
WHO: World Health Organisation
