# Supplementary material for: General anxiety, dental anxiety, digit sucking, caries and oral hygiene status of children resident in a semi-urban population in Nigeria
Source: BMC Oral Health. 2018 Apr 20;18:66. doi: 10.1186/s12903-018-0529-z (PMC5910609; doi:10.1186/s12903-018-0529-z)
Supplement: Supplementary file 1 — Individual interview schedule for children aged 0–12 years and their legal guardians. This is the comprehensive questionnaire used for the collection of data for the study. Sections 1, 3, 18 and 19 used to generate the data for this study, are accessible in the questionnaire. (DOC 1091 kb) [file 12903_2018_529_MOESM1_ESM.doc]

**A cross sectional study on the possible association between digit sucking and caries in children in Nigeria**

# INDIVIDUAL INTERVIEW SCHEDULE FOR CHILDREN AGED 0-12 YEARS AND THEIR LEGAL GUARDIANS

QUESTIONNAIRE IDENTIFICATION NUMBER |___|___|___|___|___|___|

**Introduction:** My name is…… ……… I am working for a project being implemented by a team of researchers from the Faculty of Dentistry, Obafemi Awolowo University, Ile-Ife. The study is been led by Dr KA Kolawole. We are interviewing children and their parents and care providers here in Ife Central Local Government Area in order to find out about certain oral health practices that children engage in, the causes, the consequences of the behaviour on the oral health, and find out how best to address these behaviours in this environment.

**Confidentiality and consent:** I am going to ask you questions some of which may be very personal. Your answers are completely confidential. Your name will not be written on this form, and will never be used in connection with any of the information you tell me. This study involves 1200 children and their parents from Ife Central Local Government Area of Osun State. Your honest answers to these questions will help us better understand what the oral habits in this environment are and how we can work with parents of children who practice this oral habits to stop it. Specifically, we shall be looking at factors that link oral habits with family and parental/caregiver issues; the effect of these oral habits on the oral health. The information collected from you and people like you will help us make adequate plans for clinic and community of children who have oral habits.. We would greatly appreciate your help in responding to this survey.

(Signature of interviewer certifying that informed consent has been given verbally by respondent)

Interviewer visit

|  | **Visit 1** | **Visit 2** | **Visit 3** |
| --- | --- | --- | --- |
| Date |  |  |  |
| Result |  |  |  |
| Interviewer |  |  |  |

**001 INTERVIEWERS: Code [____|____]** **Name______________ Signature________**

**002 DATE OF INTERVIEW:___\ ____ \ _____ TIME INTERVIEW STARTED_______________**

**DD MM YYYY**

**CHECKED BY SUPERVISOR__________________CODE[__]__]__] Date ____________________**

Name of Coder____________________|___|___| Signature________________ Date______________

Section 1: Background characteristics

| **No.** | **Questions and filters** | **Coding categories** | **Skip to** |
| --- | --- | --- | --- |
| Q101 | **[RECORD SEX OF THE RESPONDENT CHILD]** | Male…………….1  Female………….2 |  |
| Q102 | In what month and year were you born? | Month [___|___]  Don’t know month ………..88  Year [___|___ [___|___]  Don’t know year ………..88 |  |
| Q103 | How old were you as at your last birthday?  **[COMPARE WITH Q102 IF NEEDED AND CORRECT Q103]** | Age in completed years [___|___] |  |
| Q104 | What is your occupation i.e. what kind of work do you mainly do? | Director/upper management…………….……1  Other management……………………….……2  Sales manager/representative/Insurance Broker..3  Professional/Specialist…………………………4  Self employed/Own small business…………….5  Self employed (informal sector /hawkers/vendors etc.)…………...6  Blue collar skilled & semi skilled……………….7  Unskilled……………………………………....8  Clerk/clerical…………………………………..9  Civil Servant…………………………………10  Farmer/Forestry/Fishing/Mining……………11  Housewife……………………………………12  Pensioner/Retired……………………………13  Unemployed…………………………………14  Student……………………………………….15  Others specify[ ]…16 |  |
| Q105 | Have you ever attended school? | Yes………………….. ….. 1  No…………………. ….. 2 | **→Go to**  **Q107** |
| Q106 | What is the highest level of school you attended: Quranic only, primary, secondary or higher? | Quranic only………1  Primary …….. …..2  Secondary …….….. 3  Higher ………….. 4 |  |
| Q106A | What is the class/form/year you are currently or completed when you were in school? | Class (Primary) [___]  Form (Secondary) [___]  Year (Tertiary) [___]  Others [___] |  |

| **No**. | Questions and filters | Coding categories | Skip to |
| --- | --- | --- | --- |
| Q107 | How long have you been living continuously in this town? | Number of years [___|___]  Record 00 if less than 1 year |  |
| Q109 | What is your religion? | Islam…………. 1  Protestant.……. 2  Catholic……….. 3  Traditional.…….. 4  No religion ……. 5  Others specify.[ ]…6  No Response………9 |  |
| Q109 | How old is your mother at her last birthday?  *(please ask directly from parents)* | Age in completed years [___|___] |  |
| Q109a | What is the occupation of your mother i.e. what kind of work do you mainly do?  *(please ask directly from parents)* | Director/upper management…………….……1  Other management……………………….……2  Sales manager/representative/Insurance Broker..3  Professional/Specialist…………………………4  Self employed/Own small business…………….5  Self employed (informal sector /hawkers/vendors etc.)…………...6  Blue collar skilled & semi skilled……………….7  Unskilled……………………………………....8  Clerk/clerical…………………………………..9  Civil Servant…………………………………10  Farmer/Forestry/Fishing/Mining……………11  Housewife……………………………………12  Pensioner/Retired……………………………13  Unemployed…………………………………14  Student……………………………………….15  Others specify[ ]…16 |  |
| Q109B | What is the highest level of school your mother attended: Quranic only, primary, secondary or higher?  *(please ask directly from parents)* | Quranic only………1  Primary …….. …..2  Secondary …….….. 3  Higher ………….. 4 |  |
| Q109C | What is the class/form/year your mother completed when she was in in school?  *(please ask directly from parents)* | Class (Primary) [___]  Form (Secondary) [___]  Year (Tertiary) [___]  Others [___] |  |
| Q110 | How old is your mother at her last birthday?  *(please ask directly from parents)* | Age in completed years [___|___] |  |
| Q110a | What is the occupation of your mother i.e. what kind of work do you mainly do?  *(please ask directly from parents)* | Director/upper management…………….……1  Other management……………………….……2  Sales manager/representative/Insurance Broker..3  Professional/Specialist…………………………4  Self employed/Own small business…………….5  Self employed (informal sector /hawkers/vendors etc.)…………...6  Blue collar skilled & semi skilled……………….7  Unskilled……………………………………....8  Clerk/clerical…………………………………..9  Civil Servant…………………………………10  Farmer/Forestry/Fishing/Mining……………11  Housewife……………………………………12  Pensioner/Retired……………………………13  Unemployed…………………………………14  Student……………………………………….15  Others specify[ ]…16 |  |
| Q110B | What is the highest level of school your mother attended: Quranic only, primary, secondary or higher?  *(please ask directly from parents)* | Quranic only………1  Primary …….. …..2  Secondary …….….. 3  Higher ………….. 4 |  |
| Q110C | What is the class/form/year your mother completed when she was in in school?  *(please ask directly from parents)* | Class (Primary) [___]  Form (Secondary) [___]  Year (Tertiary) [___]  Others [___] |  |
| Q111 | To which ethnic group do you belong? | Birom ………………………………..1  Bura ………………………………….2  Edo…………………………………...3  Efik…………………………………. 4  Fulani …………………………...……5  Gwari…………………………….……6  Hausa…………………………….……7  Ibibio……………………………….…8  Idoma…………………………….….9  Igala……………………………..…….10  Igbo…………………………….…….11  Ijaw …………………………………..12  Ikwere…………………………...……13  Itsekiri………………………………..14  Kaje………………………………….15  Kanuri………………………….……16  Okrika……………………………….17  Nupe …………………………..……18  Shuwa-Arab…………………………19  Urhobo………………………………20  Tiv…………………………….……..21  Yoruba……………………………….22  Others specify[ ]..23 |  |
| Q112 | Who are you currently living with | Both Parents ……………………1  Mother only………………2  Father only………………3  Mother and stepfather …………4  Father and step mother……………5  Guardian………………….6  Cohabiting ………………….7  Room mates ………………….8  Other [ ………………] -9 |  |
| Q113 | How many of you are living in your home? | 1  2  3  4  5  6  7  8  9  10  Others…………………….99 |  |
| Q114 | How many children do your parents have? | 1  2  3  4  5  6  7  8  9  10  Others…………………….99 |  |
| Q115 | What is your position among the children your parents have? | 1  2  3  4  5  6  7  8  9  10  Others…………………….99 |  |
| Q116 | Are you one of the children of the person you are living with | Male…………….1  Female………….2  No response……………….99 |  |
| Q117 | How many meals do you eat per day?  **[READ OUT OPTIONS]** [SINGLE CODE ONLY] | Cannot guarantee one meal a day throughout the month…1  Only afford one meal a day throughout the month. 2  Only afford two meals a day throughout the month3  Afford three meals a day throughout the month…4  No Response …………………………………….5 Can afford three meals a day throughout the month …………..3 |  |

**Section 2: Infant Feeding practice**

| **No.** | **Questions and filters** | **Coding categories** | **Skip to** |
| --- | --- | --- | --- |
| Q201 | **[ASK MOTHER OF THE CHILD. IF NOT AVAILABLE THEN MOVE TO Q301]**  I would like to ask you about the feeding habits of your child [**USE NAME OF THE CHILD]**. Did you nurse this child from birth? | Yes …………………1  No ………………….2  No Response………..9 | **→Go to Q301** |
| Q201A | How old were you when you gave birth to this child [**USE NAME OF THE CHILD]**? | Years..…… [___]___]  Don’t know…………88  No response………..99 |  |
| Q202 | Did you breast feed this child [**USE NAME OF THE CHILD]**? | Yes………………….1  No…………………..2  No Response………9 | **→Go to Q207** |
| Q202a | Did you time the breast feeding of this child[**USE NAME OF THE CHILD]**? | Yes………………….1  No…………………..2  No Response………9 |  |
| Q203 | For how long did you breastfeed this child [**USE NAME OF THE CHILD]**? | Less than 1 month………….1  Less than 4 months………….2  4 to 6 months………….3  6 to 12 months………….4  12 to 18 months………….5  18 to 24 months………….6  More than 24 month………….7  Cannot remember ………….88  No Response ………….99 |  |
| Q204 | When did you first give water to the child [**USE NAME OF THE CHILD]**?? | At birth ………….1  Within 1 week of birth………….2  Within 1 month of birth………….3  Less than 4 monthsof birth………….4  4 to 6 months of birth………….5  After 6 months of birth………….6  Cannot remember ………….88  No Response ………….99 |  |
| Q205 | When did you first give solid food to the child [**USE NAME OF THE CHILD]**? | At birth ………….1  Within 1 week of birth………….2  Within 1 month of birth………….3  Less than 4 monthsof birth………….4  4 to 6 months of birth………….5  After 6 months of birth………….6  Cannot remember ………….88  No Response ………….99 |  |
| Q206 | Did you breastfeed your child in the night [**USE NAME OF THE CHILD]**? | Yes……………………………...1  No……………………….……2  No Response…………………….9 | **→Go to Q207** |
| Q206a | Do you have often leave the breast in the mouth of the child when you sleep at night? [**USE NAME OF THE CHILD]?** | Yes……………………………...1  No……………………….……2  No Response…………………….9 |  |
| Q206b | What was the reason for your stopping breastfeeding of your child [**USE NAME OF THE CHILD]**? | Had to resume work………………...1  The breast milk was not enough for the child.…2  I was advice to stop breast feeding by family…...3  I stopped for my health reasons…...4  Others specify[ ]………………...88 No Response…………………….99 | **→list (Q206c)** |

| **No.** | **Questions and filters** | **Coding categories** | **Skip to** |
| --- | --- | --- | --- |
| Q207 | Did your child use a feeder to feed [**USE NAME OF THE CHILD]**? | Yes……………………………...1  No……………………….……2  No Response…………………….9 | **→Go to Q301** |
| Q207a | Did you time the bottle feeding of this child [**USE NAME OF THE CHILD]**? | Yes……………………………...1  No……………………….……2  No Response…………………….9 |  |
| Q208 | When did your child start to use the feeding bottle [**USE NAME OF THE CHILD]**? | At birth ………….1  Within 1 week of birth………….2  Within 1 month of birth………….3  Less than 4 monthsof birth………….4  4 to 6 months of birth………….5  After 6 months of birth………….6  Cannot remember ………….88  No Response ………….99 |  |
| Q209 | Did you bottle feed your child in the night [**USE NAME OF THE CHILD]**? | Yes……………………………...1  No……………………….……2  No Response…………………….9 |  |
| Q209a | Do you have often leave the bottle in the mouth of the child when you sleep at night?  [**USE NAME OF THE CHILD]?** | Yes……………………………...1  No……………………….……2  No Response…………………….9 |  |

**Section 3: Oral habits (1) – digit and finger sucking.**

| No. | **Questions and filters** | Coding categories | Skip to |
| --- | --- | --- | --- |

| Q301 | From now on, I will ask you specific questions about possible oral habits of your child. Do/Did you child/you ever suck any of the fingers [**USE NAME OF THE CHILD]**?  [**DESCRIBE WHAT DIGIT SUCKING IS TO THE RESPONDENT**] | | Yes..……….1  No……… 2  No response ……… 99 | | | **→Go to Q401** |
| --- | --- | --- | --- | --- | --- | --- |
| Q302 | Which finger did your child/you suck?  [**USE NAME OF THE CHILD]** | | Thumb ………….….1  Digits …………..2  Cannot remember…..…..8 | | | **→Go to Q303** |
| Q302a | How many fingers did your child/you suck?  [**USE NAME OF THE CHILD]** | | 1  2  3  4  Cannot remember…..…..88 | | |  |
| Q303 | At what age did your child/you start engaging in this habit?  [**USE NAME OF THE CHILD]** | | Age in years [___|___]  **OR**  Age in months [___|___]  Child is currently still sucking…..…..3  Cannot remember…..…..88 | | |  |
| Q303a | At what age did your child/you stop engaging in this habit?  [**USE NAME OF THE CHILD]** | | Age in years [___|___]  **OR**  Age in months [___|___]  Cannot remember…..…..88 | | |  |
| Q304 | For how long did your child/you engage in this habit?  [**USE NAME OF THE CHILD]** | | Number of years [___|___]  **OR**  Number of months [___|___]  Child is currently still sucking…..…..3  Cannot remember…..…..88 | | |  |
| Q307 | How often did your child/you engaged in this habit?  [**USE NAME OF THE CHILD]** | | Irregularly………….….1  Once a week………….….2  A few (2-3) times a week………….….3  Once a day………….….4  Several times a day………….….5  Cannot remember ………….….88  No response ………….….99 | | |  |
| Q308 | Each time your child /you suck, how long does it last for?  [**USE NAME OF THE CHILD]** | | | Less than a minute………….….1  1-5 minutes………….….2  5-10 minutes………….….3  10 – 20 minutes………….….4  20 – 30 minutes………….….5  Almost continually ………….….88  No response ………….….99 | |  |
| Q308a | When does your child/you engage with the habit?  **[USE NAME OF THE CHILD]** | | | Early in the morning………….….1  Before meals ………….….2  When alone………….….3  Before bedtime………….….4  During sleep………….….5  No time pattern observed ………….….6  Cannot remember ………….….88  No response ………….….99 | |  |
| Q309 | What do you think makes your child/you engage with the habit?  **[USE NAME OF THE CHILD]** | | | When it is night time………….….1  When the child wants to breastfeed……….2  When the pacifier is not available…….….3  When mother is not around………….….4  When anxious………….….5  When hungry………….….6  Others, please mention…………………..88 | |  |
| Q310 | When your child/you suck, do you hear the sucking sound?  **[USE NAME OF THE CHILD]** | | | Yes……1  No…..2  Cannot remember ………….….88  No response ………….….99 | |  |
| Q311 | When your child/you suck, do you hear a popping sound [*please make the sound]*?  **[USE NAME OF THE CHILD]** | | | Yes……1  No…..2  Cannot remember ………….….88  No response ………….….99 | |  |
| Q312 | Did you have any concerns about your child/you habit? | | | Yes……1  No…..2  Cannot remember ………….….88  No response ………….….99 | | **→Go to Q313** |
| Q312a | What were you worried about? | | | Habit might continue until child becomes older ….1  Habit might affect shape of teeth ………….….2  Habit might affect child appearance………….….3  Habit might affect child’s in school performance ….4  Child’s friends may tease him/her……….….5  Others specify[ ]………………...88 | | **→list (q312b)** |
| Q313 | Did you seek advice from anyone about the habit? | | | Yes……1  No…..2  Cannot remember ………….….88  No response ………….….99 | |  |
| Q313a | Who did you seek advice from? | | | From friends……………...1  From religious leaders………………….2  From counsellors…………………..………….3  From medical doctor…………………..………….4  From dentists…………………..………….5  Others specify[ ]………………...88 | |  |
| Q314a | How did you *(specifically ask parents)* try to stop the habit? | | | Encouraged peer teasing……………...1  Punishing the child for sucking…………2  Application of unpleasant flavoring substance on the finger………….................................................................3  Child broke habit voluntarily…………….4  Interrupting the use of pacifier……………...5  Gave rewards for not sucking ……………...6  Wrapping the hand or tape application to the digit..7  Applying unpleasant flavoring substance on the pacifier…………..............................................................8  Using a dental appliance……………...9  Others specify[ ]………………...88 | |  |
| Q314b | How did you *(specifically ask the child if can communicate)* try to stop the habit? | | | Peer teasing……………...1  Punishment from parents…………2  Application of unpleasant flavoring substance on the finger………….................................................................3  Child broke habit voluntarily…………….4  Received rewards for not sucking ……………...5  Wrapped the hand/finger to prevent sucking..6  Using a dental appliance……………...7  Others specify[ ]………………...88 | |  |
| Q315 | Which effort(s) did you think worked? | Encouraged peer teasing……………...1  Punishing the child for sucking…………2  Application of unpleasant flavoring substance on the finger…………...............................................................3  Child broke habit voluntarily……….4  Interrupting the use of pacifier……………...5  Gave rewards for not sucking ……………...6  Wrapping the hand or tape application to the digit..7  Applying unpleasant flavoring substance on the pacifier…………............................................................8  Using a dental appliance……………...9  Others specify[ ]…………...88 | | |  | |
| Q315a | Which effort did you think did not worked? | Encouraged peer teasing……………...1  Punishing the child for sucking…………2  Application of unpleasant flavoring substance on the finger…………...............................................................3  Child broke habit voluntarily…….4  Interrupting the use of pacifier……………...5  Gave rewards for not sucking ……………...6  Wrapping the hand or tape application to the digit..7  Applying unpleasant flavoring substance on the pacifier…………............................................................8  Using a dental appliance……………...9  Others specify[ ]……………...88 | | |  | |

**Section 4: Oral habits (2) – tongue sucking**

| No. | **Questions and filters** | Coding categories | Skip to |
| --- | --- | --- | --- |

| Q401 | Do/Did your child/you suck your tongue [**USE NAME OF THE CHILD]**?  [**DESCRIBE WHAT TONGUE SUCKING IS TO THE RESPONDENT**] | | Yes..……….1  No……… 2  No response ……… 99 | | **→Go to Q501** |
| --- | --- | --- | --- | --- | --- |
| Q402 | At what age did your child/you start engaging in this habit?  [**USE NAME OF THE CHILD]** | | Age in years [___|___]  **OR**  Age in months [___|___]  Cannot remember…..…..88 | |  |
| Q403 | For how long did your child/you engage in this habit?  [**USE NAME OF THE CHILD]** | | Number of years [___|___]  **OR**  Number of months [___|___]  Child is still sucking…..…..3  Cannot remember…..…..88 | |  |
| Q403a | At what age did your child/you stop engaging in this habit?  [**USE NAME OF THE CHILD]** | | Age in years [___|___]  **OR**  Age in months [___|___]  Cannot remember…..…..88 | |  |
| Q404 | How often did your child/you engaged in this habit?  [**USE NAME OF THE CHILD]** | | Irregularly………….….1  Once a week………….….2  A few (2-3) times a week………….….3  Once a day………….….4  Several times a day………….….5  Cannot remember ………….….88  No response ………….….99 | |  |
| Q405 | Each time your child /you suck, how long does it last for?  [**USE NAME OF THE CHILD]** | | | Less than a minute………….….1  1-5 minutes………….….2  5-10 minutes………….….3  10 – 20 minutes………….….4  20 – 30 minutes………….….5  Almost continually ………….….88  No response ………….….99 |  |
| Q406 | When does your child/you engage with the habit?  **[USE NAME OF THE CHILD]** | | | Early in the morning………….….1  Before meals ………….….2  When alone………….….3  Before bedtime………….….4  During sleep………….….5  No time pattern observed ………….….6  Cannot remember ………….….88  No response ………….….99 |  |
| Q407 | What do you think makes your child/you engage with the habit?  **[USE NAME OF THE CHILD]** | | | When it is night time………….….1  When the child wants to breastfeed……….2  When the pacifier is not available…….….3  When mother is not around………….….4  When anxious………….….5  When hungry………….….6  Others, please mention…………………..88 |  |
| Q408 | When your child/you suck, do you hear the sucking sound?  **[USE NAME OF THE CHILD]** | | | Yes……1  No…..2  Cannot remember ………….….88  No response ………….….99 |  |
| Q409 | Did you have any concerns about your child/you habit? | | | Yes……1  No…..2  Cannot remember ………….….88  No response ………….….99 | **→Go to Q410** |
| Q409a | What were you worried about? | | | Habit might continue until child becomes older ….1  Habit might affect shape of teeth ………….….2  Habit might affect child appearance………….….3  Habit might affect child’s in school performance ….4  Child’s friends may tease him/her……….….5  Others specify[ ]………………...88 | **→list (q409b)** |
| Q410 | Did you seek advice from anyone about the habit? | | | Yes……1  No…..2  Cannot remember ………….….88  No response ………….….99 |  |
| Q410a | Who did you seek advice from? | | | From friends……………...1  From religious leaders………………….2  From 16ounselors…………………..………….3  From medical doctor…………………..………….4  From dentists…………………..………….5  Others specify[ ]………………...88 |  |
| Q411a | How did you *(specifically ask parents)* try to stop the habit? | | | Encouraged peer teasing………………1  Punishing the child for sucking…………2  Application of unpleasant flavoring substance on the finger……………..............................................................3  Child broke habit voluntarily…………….4  Interrupting the use of pacifier……………...5  Gave rewards for not sucking ……………...6  Wrapping the hand or tape application to the digit..7  Applying unpleasant flavoring substance on the pacifier…………..............................................................8  Using a dental appliance……………...9  Others specify[ ]………………...88 |  |
| Q411b | How did you *(specifically ask the child if can communicate)* try to stop the habit? | | | Peer teasing………………1  Punishment from parents…………2  Application of unpleasant flavoring substance on the finger……………..............................................................3  Child broke habit voluntarily…………….4  Received rewards for not sucking ……………...5  Wrapped the hand/finger to prevent sucking..6  Using a dental appliance……………...7  Others specify[ ]………………...88 |  |
| Q412a | Which effort(s) did you think worked? | Encouraged peer teasing………………1  Punishing the child for sucking…………2  Child broke habit voluntarily……….3  Interrupting the use of pacifier……………...4  Gave rewards for not sucking ……………...5  Using a dental appliance……………...6  Others specify[ ]…………...88 | | |  |
| Q412b | Which effort did you think did not worked? | Encouraged peer teasing………………1  Punishing the child for sucking…………2  Child broke habit voluntarily……….3  Interrupting the use of pacifier……………...4  Gave rewards for not sucking ……………...5  Using a dental appliance……………...6  Others specify[ ]…………...88 | | |  |

**Section 5: Oral habits (3) – tongue thrusting**

| No. | **Questions and filters** | Coding categories | Skip to |
| --- | --- | --- | --- |

| Q501 | Do you child/you thrust your tongue [**USE NAME OF THE CHILD]**?  [**DESCRIBE WHAT TONGUE THRUSTING IS TO THE RESPONDENT**] | | Yes..……….1  No……… 2  No response ……… 99 | | | **→Go to Q601** |
| --- | --- | --- | --- | --- | --- | --- |
| Q502 | At what age did your child/you start engaging in this habit?  [**USE NAME OF THE CHILD]** | | Age in years [___|___]  **OR**  Age in months [___|___]  Cannot remember…..…..88 | | |  |
| Q503 | For how long did your child/you engage in this habit?  [**USE NAME OF THE CHILD]** | | Number of years [___|___]  **OR**  Number of months [___|___]  Child is currently still thrusting…..…..3  Cannot remember…..…..88 | | |  |
| Q503a | At what age did your child/you stop engaging in this habit?  [**USE NAME OF THE CHILD]** | | Age in years [___|___]  **OR**  Age in months [___|___]  Cannot remember…..…..88 | | |  |
| Q504 | How often did your child/you engaged in this habit?  [**USE NAME OF THE CHILD]** | | Irregularly………….….1  Once a week………….….2  A few (2-3) times a week………….….3  Once a day………….….4  Several times a day………….….5  Cannot remember ………….….88  No response ………….….99 | | |  |
| Q505 | Each time your child /you suck, how long does it last for?  [**USE NAME OF THE CHILD]** | | | Less than a minute………….….1  1-5 minutes………….….2  5-10 minutes………….….3  10 – 20 minutes………….….4  20 – 30 minutes………….….5  Almost continually ………….….88  No response ………….….99 | |  |
| Q506 | When does your child/you engage with the habit?  **[USE NAME OF THE CHILD]** | | | Early in the morning………….….1  Before meals ………….….2  When alone………….….3  Before bedtime………….….4  During sleep………….….5  No time pattern observed ………….….6  Cannot remember ………….….88  No response ………….….99 | |  |
| Q507 | What do you think makes your child/you engage with the habit?  **[USE NAME OF THE CHILD]** | | | When it is night time………….….1  When the child wants to breastfeed……….2  When the pacifier is not available…….….3  When mother is not around………….….4  When anxious………….….5  When hungry………….….6  Others, please mention…………………..88 | |  |
| Q508 | When your child/you suck, do you hear the sucking sound?  **[USE NAME OF THE CHILD]** | | | Yes……1  No…..2  Cannot remember ………….….88  No response ………….….99 | |  |
| Q509 | Did you have any concerns about your child/you habit? | | | Yes……1  No…..2  Cannot remember ………….….88  No response ………….….99 | | **→Go to Q510** |
| Q509a | What were you worried about? | | | Habit might continue until child becomes older ….1  Habit might affect shape of teeth ………….….2  Habit might affect child appearance………….….3  Habit might affect child’s in school performance ….4  Child’s friends may tease him/her……….….5  Others specify[ ]………………...88 | | **→list (q509b)** |
| Q510 | Did you seek advice from anyone about the habit? | | | Yes……1  No…..2  Cannot remember ………….….88  No response ………….….99 | |  |
| Q510a | Who did you seek advice from? | | | From friends……………...1  From religious leaders………………….2  From counsellors…………………..………….3  From medical doctor…………………..………….4  From dentists…………………..………….5  Others specify[ ]………………...88 | |  |
| Q511a | How did you *(specifically ask parents)* try to stop the habit? | | | Encouraged peer teasing……………...1  Punishing the child for sucking…………2  Application of unpleasant flavoring substance on the finger………….................................................................3  Child broke habit voluntarily…………….4  Interrupting the use of pacifier……………...5  Gave rewards for not sucking ……………...6  Wrapping the hand or tape application to the digit..7  Applying unpleasant flavoring substance on the pacifier…………..............................................................8  Using a dental appliance……………...9  Others specify[ ]………………...88 | |  |
| Q511b | How did you *(specifically ask the child if can communicate)* try to stop the habit? | | | Peer teasing……………...1  Punishment from parents…………2  Application of unpleasant flavoring substance on the finger………….................................................................3  Child broke habit voluntarily…………….4  Received rewards for not sucking ……………...5  Wrapped the hand/finger to prevent sucking..6  Using a dental appliance……………...7  Others specify[ ]………………...88 | |  |
| Q512a | Which effort(s) did you think worked? | Encouraged peer teasing……………...1  Punishing the child for sucking…………2  Child broke habit voluntarily……….3  Interrupting the use of pacifier……………...4  Gave rewards for not thrusting ……………...5  Using a dental appliance……………...6  Others specify[ ]…………...88 | | |  | |
| Q512b | Which effort did you think did not worked? | Encouraged peer teasing……………...1  Punishing the child for sucking…………2  Child broke habit voluntarily……….3  Interrupting the use of pacifier……………...4  Gave rewards for not thrusting ……………...5  Using a dental appliance……………...6  Others specify[ ]…………...88 | | |  | |

**Section 6: Oral habits (4) – lip sucking**

| No. | **Questions and filters** | Coding categories | Skip to |
| --- | --- | --- | --- |

| Q601 | Do/did you child/you suck your to lip [**USE NAME OF THE CHILD]**?  [**DESCRIBE WHAT LIP SUCKING IS TO THE RESPONDENT**] | | Yes..……….1  No……… 2  No response ……… 99 | | **→Go to Q701** |
| --- | --- | --- | --- | --- | --- |
| Q602 | At what age did your child/you start engaging in this habit?  [**USE NAME OF THE CHILD]** | | Age in years [___|___]  **OR**  Age in months [___|___]  Cannot remember…..…..88 | |  |
| Q603 | For how long did your child/you engage in this habit?  [**USE NAME OF THE CHILD]** | | Number of years [___|___]  **OR**  Number of months [___|___]  Child is currently still sucking…..…..3  Cannot remember…..…..88 | |  |
| Q603a | At what age did your child/you stop engaging in this habit?  [**USE NAME OF THE CHILD]** | | Age in years [___|___]  **OR**  Age in months [___|___]  Cannot remember…..…..88 | |  |
| Q604 | How often did your child/you engaged in this habit?  [**USE NAME OF THE CHILD]** | | Irregularly………….….1  Once a week………….….2  A few (2-3) times a week………….….3  Once a day………….….4  Several times a day………….….5  Cannot remember ………….….88  No response ………….….99 | |  |
| Q605 | Each time your child /you suck, how long does it last for?  [**USE NAME OF THE CHILD]** | | | Less than a minute………….….1  1-5 minutes………….….2  5-10 minutes………….….3  10 – 20 minutes………….….4  20 – 30 minutes………….….5  Almost continually ………….….88  No response ………….….99 |  |
| Q606 | When does your child/you engage with the habit?  **[USE NAME OF THE CHILD]** | | | Early in the morning………….….1  Before meals ………….….2  When alone………….….3  Before bedtime………….….4  During sleep………….….5  No time pattern observed ………….….6  Cannot remember ………….….88  No response ………….….99 |  |
| Q607 | What do you think makes your child/you engage with the habit?  **[USE NAME OF THE CHILD]** | | | When it is night time………….….1  When the child wants to breastfeed……….2  When the pacifier is not available…….….3  When mother is not around………….….4  When anxious………….….5  When hungry………….….6  Others, please mention…………………..88 |  |
| Q608 | When your child/you suck, do you hear the sucking sound?  **[USE NAME OF THE CHILD]** | | | Yes……1  No…..2  Cannot remember ………….….88  No response ………….….99 |  |
| Q609 | Did you have any concerns about your child/you habit? | | | Yes……1  No…..2  Cannot remember ………….….88  No response ………….….99 | **→Go to Q610** |
| Q609a | What were you worried about? | | | Habit might continue until child becomes older ….1  Habit might affect shape of teeth ………….….2  Habit might affect child appearance………….….3  Habit might affect child’s in school performance ….4  Child’s friends may tease him/her……….….5  Others specify[ ]………………...88 | **→list (q609b)** |
| Q610 | Did you seek advice from anyone about the habit? | | | Yes……1  No…..2  Cannot remember ………….….88  No response ………….….99 |  |
| Q610a | Who did you seek advice from? | | | From friends……………...1  From religious leaders………………….2  From counsellors…………………..………….3  From medical doctor…………………..………….4  From dentists…………………..………….5  Others specify[ ]………………...88 |  |
| Q611a | How did you *(specifically ask parents)* try to stop the habit? | | | Encouraged peer teasing……………...1  Punishing the child for sucking…………2  Application of unpleasant flavoring substance on the lip….………….................................................................3  Child broke habit voluntarily…………….4  Interrupting the use of pacifier……………...5  Gave rewards for not sucking ……………...6  Using a dental appliance……………...7  Others specify[ ]………………...88 |  |
| Q611b | How did you *(specifically ask the child if can communicate)* try to stop the habit? | | | Peer teasing……………...1  Punishment from parents…………2  Application of unpleasant flavoring substance on the lip……………...................................................................3  Child broke habit voluntarily…………….4  Received rewards for not sucking ……………...5  Using a dental appliance……………...6  Others specify[ ]………………...88 |  |
| Q612a | Which effort(s) did you think worked? | Encouraged peer teasing……………...1  Punishing the child for sucking…………2  Child broke habit voluntarily……….3  Interrupting the use of pacifier……………...4  Gave rewards for not sucking ……………...5  Using a dental appliance……………...6  Others specify[ ]…………...88 | | |  |
| Q612b | Which effort did you think did not worked? | Encouraged peer teasing……………...1  Punishing the child for sucking…………2  Child broke habit voluntarily……….3  Interrupting the use of pacifier……………...4  Gave rewards for not sucking ……………...5  Using a dental appliance……………...6  Others specify[ ]…………...88 | | |  |

**Section 7: Oral habits (5) – lip biting**

| No. | **Questions and filters** | Coding categories | Skip to |
| --- | --- | --- | --- |

| Q701 | Do/Did you child/you bite your to lip [**USE NAME OF THE CHILD]**?  [**DESCRIBE WHAT LIP BITING IS TO THE RESPONDENT**] | | Yes..……….1  No……… 2  No response ……… 99 | | **→Go to Q801** |
| --- | --- | --- | --- | --- | --- |
| Q702 | At what age did your child/you start engaging in this habit?  [**USE NAME OF THE CHILD]** | | Age in years [___|___]  **OR**  Age in months [___|___]  Cannot remember…..…..88 | |  |
| Q703 | For how long did your child/you engage in this habit?  [**USE NAME OF THE CHILD]** | | Number of years [___|___]  **OR**  Number of months [___|___]  Child is currently still biting…..…..3  Cannot remember…..…..88 | |  |
| Q703a | At what age did your child/you stop engaging in this habit?  [**USE NAME OF THE CHILD]** | | Age in years [___|___]  **OR**  Age in months [___|___]  Cannot remember…..…..88 | |  |
| Q704 | How often did your child/you engaged in this habit?  [**USE NAME OF THE CHILD]** | | Irregularly………….….1  Once a week………….….2  A few (2-3) times a week………….….3  Once a day………….….4  Several times a day………….….5  Cannot remember ………….….88  No response ………….….99 | |  |
| Q605 | Each time your child /you suck, how long does it last for?  [**USE NAME OF THE CHILD]** | | | Less than a minute………….….1  1-5 minutes………….….2  5-10 minutes………….….3  10 – 20 minutes………….….4  20 – 30 minutes………….….5  Almost continually ………….….88  No response ………….….99 |  |
| Q706 | When does your child/you engage with the habit?  **[USE NAME OF THE CHILD]** | | | Early in the morning………….….1  Before meals ………….….2  When alone………….….3  Before bedtime………….….4  During sleep………….….5  No time pattern observed ………….….6  Cannot remember ………….….88  No response ………….….99 |  |
| Q707 | What do you think makes your child/you engage with the habit?  **[USE NAME OF THE CHILD]** | | | When it is night time………….….1  When the child wants to breastfeed……….2  When the pacifier is not available…….….3  When mother is not around………….….4  When hungry………….….5  When anxious………….….6  Others, please mention…………………..88 |  |
| Q708 | When your child/you bits the lips, does it bleed?  **[USE NAME OF THE CHILD]** | | | Yes……1  No…..2  Cannot remember ………….….88  No response ………….….99 |  |
| Q709 | Did you have any concerns about your child/you habit? | | | Yes……1  No…..2  Cannot remember ………….….88  No response ………….….99 | **→Go to Q710** |
| Q709a | What were you worried about? | | | Habit might continue until child becomes older ….1  Habit might affect shape of teeth ………….….2  Habit might affect child appearance………….….3  Habit might affect child’s in school performance ….4  Child’s friends may tease him/her……….….5  Others specify[ ]………………...88 | **→list (q709b)** |
| Q710 | Did you seek advice from anyone about the habit? | | | Yes……1  No…..2  Cannot remember ………….….88  No response ………….….99 |  |
| Q710a | Who did you seek advice from? | | | From friends……………...1  From religious leaders………………….2  From counsellors…………………..………….3  From medical doctor…………………..………….4  From dentists…………………..………….5  Others specify[ ]………………...88 |  |
| Q711a | How did you *(specifically ask parents)* try to stop the habit? | | | Encouraged peer teasing……………...1  Punishing the child for sucking…………2  Application of unpleasant flavoring substance on the lip….………….................................................................3  Child broke habit voluntarily…………….4  Interrupting the use of pacifier……………...5  Gave rewards for not biting ……………...6  Using a dental appliance……………...7  Others specify[ ]………………...88 |  |
| Q711b | How did you *(specifically ask the child if can communicate)* try to stop the habit? | | | Peer teasing……………...1  Punishment from parents…………2  Application of unpleasant flavoring substance on the lip……………...................................................................3  Child broke habit voluntarily…………….4  Received rewards for not biting ……………...5  Using a dental appliance……………...6  Others specify[ ]………………...88 |  |
| Q712a | Which effort(s) did you think worked? | Encouraged peer teasing……………...1  Punishing the child for sucking…………2  Child broke habit voluntarily……….3  Interrupting the use of pacifier……………...4  Gave rewards for not biting ……………...5  Using a dental appliance……………...6  Others specify[ ]…………...88 | | |  |
| Q712b | Which effort did you think did not worked? | Encouraged peer teasing……………...1  Punishing the child for sucking…………2  Child broke habit voluntarily……….3  Interrupting the use of pacifier……………...4  Gave rewards for not biting ……………...5  Using a dental appliance……………...6  Others specify[ ]…………...88 | | |  |

**Section 8: Oral habits (6) – nail biting**

| No. | **Questions and filters** | Coding categories | Skip to |
| --- | --- | --- | --- |

| Q801 | Do/Did you child/you bite your to nail [**USE NAME OF THE CHILD]**?  [**DESCRIBE WHAT NAIL BITING IS TO THE RESPONDENT**] | | Yes..……….1  No……… 2  No response ……… 99 | | **→Go to Q901** |
| --- | --- | --- | --- | --- | --- |
| Q802 | At what age did your child/you start engaging in this habit?  [**USE NAME OF THE CHILD]** | | Age in years [___|___]  **OR**  Age in months [___|___]  Cannot remember…..…..88 | |  |
| Q803 | For how long did your child/you engage in this habit?  [**USE NAME OF THE CHILD]** | | Number of years [___|___]  **OR**  Number of months [___|___]  Child is currently still biting…..…..3  Cannot remember…..…..88 | |  |
| Q803a | At what age did your child/you stop engaging in this habit?  [**USE NAME OF THE CHILD]** | | Age in years [___|___]  **OR**  Age in months [___|___]  Cannot remember…..…..88 | |  |
| Q804 | How often did your child/you engaged in this habit?  [**USE NAME OF THE CHILD]** | | Irregularly………….….1  Once a week………….….2  A few (2-3) times a week………….….3  Once a day………….….4  Several times a day………….….5  Cannot remember ………….….88  No response ………….….99 | |  |
| Q805 | Each time your child /you bite your nails, how long does it last for?  [**USE NAME OF THE CHILD]** | | | Less than a minute………….….1  1-5 minutes………….….2  5-10 minutes………….….3  10 – 20 minutes………….….4  20 – 30 minutes………….….5  Almost continually ………….….88  No response ………….….99 |  |
| Q806 | When does your child/you engage with the habit?  **[USE NAME OF THE CHILD]** | | | Early in the morning………….….1  Before meals ………….….2  When alone………….….3  Before bedtime………….….4  During sleep………….….5  No time pattern observed ………….….6  Cannot remember ………….….88  No response ………….….99 |  |
| Q807 | What do you think makes your child/you engage with the habit?  **[USE NAME OF THE CHILD]** | | | When it is night time………….….1  When the child wants to breastfeed……….2  When the pacifier is not available…….….3  When mother is not around………….….4  When hungry………….….5  When anxious………….….6  Others, please mention…………………..88 |  |
| Q808 | When your child/you bits the nails, does the fingers bleed?  **[USE NAME OF THE CHILD]** | | | Yes……1  No…..2  Cannot remember ………….….88  No response ………….….99 |  |
| Q809 | Did you have any concerns about your child/you habit? | | | Yes……1  No…..2  Cannot remember ………….….88  No response ………….….99 | **→Go to Q810** |
| Q809a | What were you worried about? | | | Habit might continue until child becomes older ….1  Habit might affect shape of teeth ………….….2  Habit might affect shape of finger ………….….3  Habit might affect child appearance………….….4  Habit might affect child’s in school performance ….5  Child’s friends may tease him/her……….….6  Others specify[ ]………………...88 | **→list (q809b)** |
| Q810 | Did you seek advice from anyone about the habit? | | | Yes……1  No…..2  Cannot remember ………….….88  No response ………….….99 |  |
| Q810a | Who did you seek advice from? | | | From friends……………...1  From religious leaders………………….2  From counsellors…………………..………….3  From medical doctor…………………..………….4  From dentists…………………..………….5  Others specify[ ]………………...88 |  |
| Q811a | How did you *(specifically ask parents)* try to stop the habit? | | | Encouraged peer teasing……………...1  Punishing the child for biting…………2  Application of unpleasant flavoring substance on the nail….………….................................................................3  Child broke habit voluntarily…………….4  Interrupting the use of pacifier……………...5  Gave rewards for not biting ……………...6  Using a dental appliance……………...7  Others specify[ ]………………...88 |  |
| Q811b | How did you *(specifically ask the child if can communicate)* try to stop the habit? | | | Peer teasing……………...1  Punishment from parents…………2  Application of unpleasant flavoring substance on the nail…………...................................................................3  Child broke habit voluntarily…………….4  Received rewards for not biting ……………...5  Using a dental appliance……………...6  Others specify[ ]………………...88 |  |
| Q812a | Which effort(s) did you think worked? | Encouraged peer teasing……………...1  Punishing the child for biting…………2  Child broke habit voluntarily……….3  Interrupting the use of pacifier……………...4  Gave rewards for not biting ……………...5  Using a dental appliance……………...6  Others specify[ ]…………...88 | | |  |
| Q812b | Which effort did you think did not worked? | Encouraged peer teasing……………...1  Punishing the child for biting…………2  Child broke habit voluntarily……….3  Interrupting the use of pacifier……………...4  Gave rewards for not biting ……………...5  Using a dental appliance……………...6  Others specify[ ]…………...88 | | |  |

**Section 9: Oral habits (7) – object biting**

| No. | **Questions and filters** | Coding categories | Skip to |
| --- | --- | --- | --- |

| Q901 | Do/Did you child/you bite objects [**USE NAME OF THE CHILD]**?  [**DESCRIBE WHAT OBJECT BITING IS TO THE RESPONDENT**] | | Yes..……….1  No……… 2  No response ……… 99 | | **→Go to Q1001** |
| --- | --- | --- | --- | --- | --- |
| Q901a | What objects did your child/you bite?  [**USE NAME OF THE CHILD]**? | | Pen/pencil..……….1  Clothings ……… 2  Hair ……… 3  Needle/toothpick ……… 4  Others specify[ ]…………...88 | |  |
| Q902 | At what age did your child/you start engaging in this habit?  [**USE NAME OF THE CHILD]** | | Age in years [___|___]  **OR**  Number of months [___|___]  Cannot remember…..…..88 | |  |
| Q903 | For how long did your child/you engage in this habit?  [**USE NAME OF THE CHILD]** | | Age in years [___|___]  **OR**  Age in months [___|___]  Child is currently still biting…..…..3  Cannot remember…..…..88 | |  |
| Q903a | At what age did your child/you stop engaging in this habit?  [**USE NAME OF THE CHILD]** | | Age in years [___|___]  **OR**  Age in months [___|___]  Cannot remember…..…..88 | |  |
| Q904 | How often did your child/you engaged in this habit?  [**USE NAME OF THE CHILD]** | | Irregularly………….….1  Once a week………….….2  A few (2-3) times a week………….….3  Once a day………….….4  Several times a day………….….5  Cannot remember ………….….88  No response ………….….99 | |  |
| Q905 | Each time your child /you bite your nails, how long does it last for?  [**USE NAME OF THE CHILD]** | | | Less than a minute………….….1  1-5 minutes………….….2  5-10 minutes………….….3  10 – 20 minutes………….….4  20 – 30 minutes………….….5  Almost continually ………….….88  No response ………….….99 |  |
| Q906 | When does your child/you engage with the habit?  **[USE NAME OF THE CHILD]** | | | Early in the morning………….….1  Before meals ………….….2  When alone………….….3  Before bedtime………….….4  During sleep………….….5  No time pattern observed ………….….6  Cannot remember ………….….88  No response ………….….99 |  |
| Q907 | What do you think makes your child/you engage with the habit?  **[USE NAME OF THE CHILD]** | | | When it is night time………….….1  When the child wants to breastfeed……….2  When the pacifier is not available…….….3  When mother is not around………….….4  When hungry………….….5  When anxious………….….6  Others, please mention…………………..88 |  |
| Q908 | When your child/you bits the nails, does the fingers bleed?  **[USE NAME OF THE CHILD]** | | | Yes……1  No…..2  Cannot remember ………….….88  No response ………….….99 |  |
| Q909 | Did you have any concerns about your child/you habit? | | | Yes……1  No…..2  Cannot remember ………….….88  No response ………….….99 | **→Go to Q910** |
| Q909a | What were you worried about? | | | Habit might continue until child becomes older ….1  Habit might affect shape of teeth ………….….2  Habit might affect shape of finger ………….….3  Habit might affect child appearance………….….4  Habit might affect child’s in school performance ….5  Child’s friends may tease him/her……….….6  Others specify[ ]………………...88 | **→list (q909b)** |
| Q910 | Did you seek advice from anyone about the habit? | | | Yes……1  No…..2  Cannot remember ………….….88  No response ………….….99 |  |
| Q910a | Who did you seek advice from? | | | From friends……………...1  From religious leaders………………….2  From counsellors…………………..………….3  From medical doctor…………………..………….4  From dentists…………………..………….5  Others specify[ ]………………...88 |  |
| Q911a | How did you *(specifically ask parents)* try to stop the habit? | | | Encouraged peer teasing……………...1  Punishing the child for biting…………2  Application of unpleasant flavoring substance on the nail….………….................................................................3  Child broke habit voluntarily…………….4  Interrupting the use of pacifier……………...5  Gave rewards for not biting ……………...6  Using a dental appliance……………...7  Others specify[ ]………………...88 |  |
| Q911b | How did you *(specifically ask the child if can communicate)* try to stop the habit? | | | Peer teasing……………...1  Punishment from parents…………2  Application of unpleasant flavoring substance on the nail…………...................................................................3  Child broke habit voluntarily…………….4  Received rewards for not biting ……………...5  Using a dental appliance……………...6  Others specify[ ]………………...88 |  |
| Q912a | Which effort(s) did you think worked? | Encouraged peer teasing……………...1  Punishing the child for biting…………2  Child broke habit voluntarily……….3  Interrupting the use of pacifier……………...4  Gave rewards for not biting ……………...5  Using a dental appliance……………...6  Others specify[ ]…………...88 | | |  |
| Q912b | Which effort did you think did not worked? | Encouraged peer teasing……………...1  Punishing the child for biting…………2  Child broke habit voluntarily……….3  Interrupting the use of pacifier……………...4  Gave rewards for not biting ……………...5  Using a dental appliance……………...6  Others specify[ ]…………...88 | | |  |

**Section 10: Oral habits (8) – bruxism (teeth grinding).**

| No. | **Questions and filters** | Coding categories | Skip to |
| --- | --- | --- | --- |

| Q1001 | Did you child/you chew your teeth [**USE NAME OF THE CHILD]**?  [**DESCRIBE WHAT BRUXISM IS TO THE RESPONDENT**] | | Yes..……….1  No……… 2  No response ……… 99 | | **→Go to Q1101** |
| --- | --- | --- | --- | --- | --- |
| Q1002 | At what age did your child/you start engaging in this habit?  [**USE NAME OF THE CHILD]** | | Age in years [___|___]  **OR**  Number of months [___|___]  Cannot remember…..…..88 | |  |
| Q1003 | For how long did your child/you engage in this habit?  [**USE NAME OF THE CHILD]** | | Age in years [___|___]  **OR**  Age in months [___|___]  Child is currently still grinding…..…..3  Cannot remember…..…..88 | |  |
| Q1003a | At what age did your child/you stop engaging in this habit?  [**USE NAME OF THE CHILD]** | | Age in years [___|___]  **OR**  Age in months [___|___]  Cannot remember…..…..88 | |  |
| Q1004 | How often did your child/you engaged in this habit?  [**USE NAME OF THE CHILD]** | | Irregularly………….….1  Once a week………….….2  A few (2-3) times a week………….….3  Once a day………….….4  Several times a day………….….5  Cannot remember ………….….88  No response ………….….99 | |  |
| Q1005 | Each time your child /you chew the teeth, how long does it last for?  [**USE NAME OF THE CHILD]** | | | Less than a minute………….….1  1-5 minutes………….….2  5-10 minutes………….….3  10 – 20 minutes………….….4  20 – 30 minutes………….….5  Almost continually ………….….88  No response ………….….99 |  |
| Q1006 | When does your child/you engage with the habit?  **[USE NAME OF THE CHILD]** | | | Early in the morning………….….1  Before meals ………….….2  When alone………….….3  Before bedtime………….….4  During sleep………….….5  No time pattern observed ………….….6  Cannot remember ………….….88  No response ………….….99 |  |
| Q1007 | What do you think makes your child/you engage with the habit?  **[USE NAME OF THE CHILD]** | | | When it is night time………….….1  When the child wants to breastfeed……….2  When the pacifier is not available…….….3  When mother is not around………….….4  When hungry………….….5  When anxious………….….6  Others, please mention…………………..88 |  |
| Q1008 | When your child/you grind the teeth, do you hear the sound?  **[USE NAME OF THE CHILD]** | | | Yes……1  No…..2  Cannot remember ………….….88  No response ………….….99 |  |
| Q1009 | Did you have any concerns about your child/you habit? | | | Yes……1  No…..2  Cannot remember ………….….88  No response ………….….99 | **→Go to Q1010** |
| Q1009a | What were you worried about? | | | Habit might continue until child becomes older ….1  Habit might affect shape of teeth ………….….2  Habit might affect shape of finger ………….….3  Habit might affect child appearance………….….4  Habit might affect child’s in school performance ….5  Child’s friends may tease him/her……….….6  Others specify[ ]………………...88 | **→list (q1009b)** |
| Q1010 | Did you seek advice from anyone about the habit? | | | Yes……1  No…..2  Cannot remember ………….….88  No response ………….….99 |  |
| Q1010a | Who did you seek advice from? | | | From friends……………...1  From religious leaders………………….2  From counsellors…………………..………….3  From medical doctor…………………..………….4  From dentists…………………..………….5  Others specify[ ]………………...88 |  |
| Q1011a | How did you *(specifically ask parents)* try to stop the habit? | | | Encouraged peer teasing……………...1  Punishing the child for biting…………2  Child broke habit voluntarily…………….3  Interrupting the use of pacifier……………...4  Gave rewards for not biting ……………...5  Using a dental appliance……………...6  Others specify[ ]………………...88 |  |
| Q1011b | How did you *(specifically ask the child if can communicate)* try to stop the habit? | | | Peer teasing……………...1  Punishment from parents…………2  Child broke habit voluntarily…………….3  Received rewards for not biting ……………...4  Using a dental appliance……………...5  Others specify[ ]………………...88 |  |
| Q1012a | Which effort(s) did you think worked? | Encouraged peer teasing……………...1  Punishing the child for biting…………2  Child broke habit voluntarily……….3  Interrupting the use of pacifier……………...4  Gave rewards for not biting ……………...5  Using a dental appliance……………...6  Others specify[ ]…………...88 | | |  |
| Q1012b | Which effort did you think did not worked? | Encouraged peer teasing……………...1  Punishing the child for biting…………2  Child broke habit voluntarily……….3  Interrupting the use of pacifier……………...4  Gave rewards for not biting ……………...5  Using a dental appliance……………...6  Others specify[ ]…………...88 | | |  |

**Section 11: Knowledge of caries prevention**

**To be filled by mother**

| No. | **Questions and filters** | Coding categories | Skip to |
| --- | --- | --- | --- |
| I will be asking you a few questions about what you know on how to care for oral health. All your responses are confidential. I will be depending on your truthful statements to make plans for planning for children in this environment. Please do not guess. Also, do feel free to ask about things you do not understand. Thank you. | | | |

| Q1101 | Fluoridation of drinking water is an effective, safe, and efficient way to prevent holes from forming on the teeth | Strongly agree………….….1  Agree………….….2  Disagree………….….3  Strongly disagree………….….4  Don't know………….….5 | |  |
| --- | --- | --- | --- | --- |
| Q1102 | Use of fluoride containing toothpaste is an effective, safe, and efficient way to prevent holes from forming on the teeth. | Strongly agree………….….1  Agree………….….2  Disagree………….….3  Strongly disagree………….….4  Don't know………….….5 | |  |
| Q1103 | The number of times you eat sugar containing food has a great role in producing holes in the teeth | Strongly agree………….….1  Agree………….….2  Disagree………….….3  Strongly disagree………….….4  Don't know………….….5 | |  |
| Q1104 | Fissure sealant is effective in the prevention of holes developing in newly erupted molars | Strongly agree………….….1  Agree………….….2  Disagree………….….3  Strongly disagree………….….4  Don't know………….….5 | |  |
| Q1105 | Rinsing teeth with a lower amount of water after tooth-brushing increases the effect of fluoride | Strongly agree………….….1  Agree………….….2  Disagree………….….3  Strongly disagree………….….4  Don't know………….….5 | |  |
| Q1106 | Using fluoride toothpaste is more important than the brushing *per se* for preventing holes from forming on the teeth. | Strongly agree………….….1  Agree………….….2  Disagree………….….3  Strongly disagree………….….4  Don't know………….….5 | |  |
| Q1107 | Brushing twice daily with fluoride containing toothpaste is effective for preventing holes from developing in the teeth | | Strongly agree………….….1  Agree………….….2  Disagree………….….3  Strongly disagree………….….4  Don't know………….….5 |  |
| Q1108 | It is important to visit the dental clinic regularly as a measure for preventing holes from forming in the teeth. | | Strongly agree………….….1  Agree………….….2  Disagree………….….3  Strongly disagree………….….4  Don't know………….….5 |  |

**Section 12: Knowledge of caries prevention**

**To be filled by father**

| No. | **Questions and filters** | Coding categories | Skip to |
| --- | --- | --- | --- |
| I will be asking you a few questions about what you know on how to care for oral health. All your responses are confidential. I will be depending on your truthful statements to make plans for planning for children in this environment. Please do not guess. Also, do feel free to ask about things you do not understand. Thank you. | | | |

| Q1201 | Fluoridation of drinking water is an effective, safe, and efficient way to prevent holes from forming on the teeth | Strongly agree………….….1  Agree………….….2  Disagree………….….3  Strongly disagree………….….4  Don't know………….….5 | |  |
| --- | --- | --- | --- | --- |
| Q1202 | Use of fluoride containing toothpaste is an effective, safe, and efficient way to prevent holes from forming on the teeth. | Strongly agree………….….1  Agree………….….2  Disagree………….….3  Strongly disagree………….….4  Don't know………….….5 | |  |
| Q1203 | The number of times you eat sugar containing food has a great role in producing holes in the teeth | Strongly agree………….….1  Agree………….….2  Disagree………….….3  Strongly disagree………….….4  Don't know………….….5 | |  |
| Q1204 | Fissure sealant is effective in the prevention of holes developing in newly erupted molars | Strongly agree………….….1  Agree………….….2  Disagree………….….3  Strongly disagree………….….4  Don't know………….….5 | |  |
| Q1205 | Rinsing teeth with a lower amount of water after tooth-brushing increases the effect of fluoride | Strongly agree………….….1  Agree………….….2  Disagree………….….3  Strongly disagree………….….4  Don't know………….….5 | |  |
| Q1206 | Using fluoride toothpaste is more important than the brushing *per se* for preventing holes from forming on the teeth. | Strongly agree………….….1  Agree………….….2  Disagree………….….3  Strongly disagree………….….4  Don't know………….….5 | |  |
| Q1207 | Brushing twice daily with fluoride containing toothpaste is effective for preventing holes from developing in the teeth | | Strongly agree………….….1  Agree………….….2  Disagree………….….3  Strongly disagree………….….4  Don't know………….….5 |  |
| Q1208 | It is important to visit the dental clinic regularly as a measure for preventing holes from forming in the teeth. | | Strongly agree………….….1  Agree………….….2  Disagree………….….3  Strongly disagree………….….4  Don't know………….….5 |  |

**Section 13: Knowledge of caries prevention**

**To be filled by child (8 to 12 years old)**

| No. | **Questions and filters** | Coding categories | Skip to |
| --- | --- | --- | --- |
| I will be asking you a few questions about what you know on how to care for oral health. All your responses are confidential. I will be depending on your truthful statements to make plans for planning for children in this environment. Please do not guess. Also, do feel free to ask about things you do not understand. Thank you. | | | |

| Q1301 | Fluoridation of drinking water is an effective, safe, and efficient way to prevent holes from forming on the teeth | Strongly agree………….….1  Agree………….….2  Disagree………….….3  Strongly disagree………….….4  Don't know………….….5 | |  |
| --- | --- | --- | --- | --- |
| Q1302 | Use of fluoride containing toothpaste is an effective, safe, and efficient way to prevent holes from forming on the teeth. | Strongly agree………….….1  Agree………….….2  Disagree………….….3  Strongly disagree………….….4  Don't know………….….5 | |  |
| Q1303 | The number of times you eat sugar containing food has a great role in producing holes in the teeth | Strongly agree………….….1  Agree………….….2  Disagree………….….3  Strongly disagree………….….4  Don't know………….….5 | |  |
| Q1304 | Fissure sealant is effective in the prevention of holes developing in newly erupted molars | Strongly agree………….….1  Agree………….….2  Disagree………….….3  Strongly disagree………….….4  Don't know………….….5 | |  |
| Q1305 | Rinsing teeth with a lower amount of water after tooth-brushing increases the effect of fluoride | Strongly agree………….….1  Agree………….….2  Disagree………….….3  Strongly disagree………….….4  Don't know………….….5 | |  |
| Q1306 | Using fluoride toothpaste is more important than the brushing *per se* for preventing holes from forming on the teeth. | Strongly agree………….….1  Agree………….….2  Disagree………….….3  Strongly disagree………….….4  Don't know………….….5 | |  |
| Q1307 | Brushing twice daily with fluoride containing toothpaste is effective for preventing holes from developing in the teeth | | Strongly agree………….….1  Agree………….….2  Disagree………….….3  Strongly disagree………….….4  Don't know………….….5 |  |
| Q1308 | It is important to visit the dental clinic regularly as a measure for preventing holes from forming in the teeth. | | Strongly agree………….….1  Agree………….….2  Disagree………….….3  Strongly disagree………….….4  Don't know………….….5 |  |

**Section 14: Oral Health Behaviour**

**To be filled by Mother**

| No. | **Questions and filters** | Coding categories | Skip to |
| --- | --- | --- | --- |

| Q1401 | How often do you usually brush your teeth? | Irregularly or never………….….1  Once a week………….….2  A few (2-3) times a week………….….3  Once a day………….….4  Twice a day………….….5  More than twice a day………….….6  No response………….….99 | |  |
| --- | --- | --- | --- | --- |
| Q1402 | How often do you use toothpaste containing fluoride when brushing? | Always………….….1  Quiet often………….….2  Seldom………….….3  Not at all………….….4  No response………….….99 | |  |
| Q1403 | How often do you floss your teeth? | Irregularly or never………….….1  Once a week………….….2  A few (2-3) times a week………….….3  Once a day………….….4  Twice a day………….….5  More than twice a day………….….6  No response………….….99 | |  |
| Q1404 | How often do you eat sugar-containing snacks or drinks between your main meals? | About 3 times a day or more ………….….1  About twice a day ………….….2  About once a day ………….….3  Occasionally; not every day ………….….4  Rarely or never eat between meals ………….….5  No response………….….99 | |  |
| Q1405 | What do you do for your dental check-ups? | I go to a dentist to do that………….….1  I ask my or colleagues to do if for me………….….2  I do it myself………….….3  There is no need to attend dental check-ups………4  No response………….….99 | |  |
| Q1406 | When was your last dental check-up? | Within the last 6 months ………….….1  More than 6 months to one year ago ………….….2  More than 1 to 2 years ago ………….….3  More than 2 to 5 years ago ………….….4  More than 5 years ago ………….….5  Never ………….….6  Do not remember ………….….88  No response………….….99 | |  |
| Q1407 | **Do you smoke cigarettes?** | | No, never ………….….1  No, I used to, but I quit ………….….2  Yes, once a month or less ………….….3  Yes, a few times (2-3) a month ………….….4  Yes, a few times (2-3) a week ………….….5  Yes, once a day or more ………….….6  No response………….….99 |  |

**Section 15: Oral Health Behaviour**

**To be filled by Father**

| No. | **Questions and filters** | Coding categories | Skip to |
| --- | --- | --- | --- |

| Q1501 | How often do you usually brush your teeth? | Irregularly or never………….….1  Once a week………….….2  A few (2-3) times a week………….….3  Once a day………….….4  Twice a day………….….5  More than twice a day………….….6  No response………….….99 | |  |
| --- | --- | --- | --- | --- |
| Q1502 | How often do you use toothpaste containing fluoride when brushing? | Always………….….1  Quiet often………….….2  Seldom………….….3  Not at all………….….4  No response………….….99 | |  |
| Q1503 | How often do you floss your teeth? | Irregularly or never………….….1  Once a week………….….2  A few (2-3) times a week………….….3  Once a day………….….4  Twice a day………….….5  More than twice a day………….….6  No response………….….99 | |  |
| Q1504 | How often do you eat sugar-containing snacks or drinks between your main meals? | About 3 times a day or more ………….….1  About twice a day ………….….2  About once a day ………….….3  Occasionally; not every day ………….….4  Rarely or never eat between meals ………….….5  No response………….….99 | |  |
| Q1505 | What do you do for your dental check-ups? | I go to a dentist to do that………….….1  I ask my or colleagues to do if for me………….….2  I do it myself………….….3  There is no need to attend dental check-ups………4  No response………….….99 | |  |
| Q1506 | When was your last dental check-up? | Within the last 6 months ………….….1  More than 6 months to one year ago ………….….2  More than 1 to 2 years ago ………….….3  More than 2 to 5 years ago ………….….4  More than 5 years ago ………….….5  Never ………….….6  Do not remember ………….….88  No response………….….99 | |  |
| Q1507 | **Do you smoke cigarettes?** | | No, never ………….….1  No, I used to, but I quit ………….….2  Yes, once a month or less ………….….3  Yes, a few times (2-3) a month ………….….4  Yes, a few times (2-3) a week ………….….5  Yes, once a day or more ………….….6  No response………….….99 |  |

**Section 16: Oral Health Behaviour**

**To be filled by Child**

| No. | **Questions and filters** | Coding categories | Skip to |
| --- | --- | --- | --- |

| Q1601 | How often do you usually brush your teeth? | Irregularly or never………….….1  Once a week………….….2  A few (2-3) times a week………….….3  Once a day………….….4  Twice a day………….….5  More than twice a day………….….6  No response………….….99 | |  |
| --- | --- | --- | --- | --- |
| Q1602 | When do you brush your teeth?  *Read the question and tick all that are applicable. Do not read the options* | Morning before breakfast………….….1  Morning after breakfast………….….2  Afternoon before lunch………….….3  Afternoon after lunch………….….4  Evening before supper………….….5  Evening after supper………….….6  No regular time intervals………….….7  No response………….….99 | |  |
| Q1603 | Who brushes your teeth for you? | Myself ………….….1  My mum/guardian brushes for me every day………2  My mum/guardian brushes for me once a week……3  My mum/guardian brushes for me occasionally……4  Others …………………………………………99 | |  |
| Q1604 | What do you use to brush your teeth?  *Read the question and tick all that are applicable. Do not read the options* | Toothbrush ………….….1  Chewing stick………….….2  Salt ………….….3  Cotton wool………….….4  Towel ………….….5  Charcoal ………….….6  Ground glass………….….7  Others …………………………………………88  No response………….….99 | |  |
| Q1605 | How often do you use toothpaste containing fluoride when brushing? | Always………….….1  Quiet often………….….2  Seldom………….….3  Not at all………….….4  No response………….….99 | |  |
| Q1606 | How often do you use mouth rinses? | Always………….….1  Quiet often………….….2  Seldom………….….3  Never………….….4  No response………….….99 | |  |
| Q1607 | How often do you use topical fluorides? | Always………….….1  Quiet often………….….2  Seldom………….….3  Never………….….4  No response………….….99 | |  |
| Q1608 | How often do you floss your teeth? | Irregularly or never………….….1  Once a week………….….2  A few (2-3) times a week………….….3  Once a day………….….4  Twice a day………….….5  More than twice a day………….….6  No response………….….99 | |  |
| Q1609 | How often do you eat sugar-containing snacks or drinks between your main meals? | About 3 times a day or more ………….….1  About twice a day ………….….2  About once a day ………….….3  Occasionally; not every day ………….….4  Rarely or never eat between meals ………….….5  No response………….….99 | |  |
| Q1610 | What do you do for your dental check-ups? | I go to a dentist to do that………….….1  I ask my or colleagues to do if for me………….….2  I do it myself………….….3  There is no need to attend dental check-ups………4  No response………….….99 | |  |
| Q1611 | When was your last dental check-up? | Within the last 6 months ………….….1  More than 6 months to one year ago ………….….2  More than 1 to 2 years ago ………….….3  More than 2 to 5 years ago ………….….4  More than 5 years ago ………….….5  Never ………….….6  Do not remember ………….….88  No response………….….99 | |  |
| Q1612 | Do you smoke cigarettes? | | No, never ………….….1  No, I used to, but I quit ………….….2  Yes, once a month or less ………….….3  Yes, a few times (2-3) a month ………….….4  Yes, a few times (2-3) a week ………….….5  Yes, once a day or more ………….….6  No response………….….99 |  |

**Section 17: Medical and Dietary History of the Child**

**To be filled by mother for the Child**

| No. | **Questions and filters** | Coding categories | Skip to |
| --- | --- | --- | --- |
| We will be asking a few details about the health of the child. Once again, all your responses are confidential. Feel free to ask questions about what you are not certain about. Please respond as truthfully as possible and where you do not have answers, please do lets know. | | | |

| Q1701 | Does your child vomit excessively? | Yes ………….….1  No ………….….2  Don’t know………….….88  No response………….….99 | |  |
| --- | --- | --- | --- | --- |
| Q1702 | Does your child have a eating disorder? | Yes ………….….1  No ………….….2  Don’t know………….….88  No response………….….99 | |  |
| Q1703 | Does the child regularly brings back food from the stomach into the mouth? | Yes ………….….1  No ………….….2  Don’t know………….….88  No response………….….99 | |  |
| Q1704 | Does the child regularly use medicines for stomach ulcer? | Yes ………….….1  No ………….….2  Don’t know………….….88  No response………….….99 | |  |
| Q1705 | Does the child take lots of alcohol? | Yes ………….….1  No ………….….2  Don’t know………….….88  No response………….….99 | |  |
| Q1706 | Does the child have problems that cause the eyes and the skin to be very dry and do not produce sweat or tears? | Yes ………….….1  No ………….….2  Don’t know………….….88  No response………….….99 | |  |
| Q1707 | Has the child ever had cancer in the head and neck that caused him/her to have radiotherapy of the head and neck? | Yes ………….….1  No ………….….2  Don’t know………….….88  No response………….….99 | |  |
| Q1708 | Does the child use drugs that make the mouth dry? | Yes ………….….1  No ………….….2  Don’t know………….….88  No response………….….99 | |  |
| Q1709 | Does the child take a lot of fruit juices and bottled drinks – coke etc | Yes ………….….1  No ………….….2  Don’t know………….….88  No response………….….99 | |  |
| Q1710 | Does the child put the food in the mouth for a long time before swallowing? | | Yes ………….….1  No ………….….2  Don’t know………….….88  No response………….….99 |  |
| Q1711 | Does the child spend lots of time around a battery charger? | | Yes ………….….1  No ………….….2  Don’t know………….….88  No response………….….99 |  |

Q1712

Draw a dietary chart for 2 days recall and one present day

**Section 18**

**CFSS-DS**

**To be filled by parents of children 6-7 years or those with poor communication skills**

**To be filled by children 8-12 years old**

Estimate the level of fear your son/daughter would perceive in the situations described below. Mark your answer with X.

How afraid is your son or daughter of …………

|  |  | **Not afraid at all** | **A little afraid** | **A fair amount** | **Pretty much afraid** | **Very afraid** |
| --- | --- | --- | --- | --- | --- | --- |
| 1801 | Dentists |  |  |  |  |  |
| 1802 | Doctors |  |  |  |  |  |
| 1803 | Injections |  |  |  |  |  |
| 1804 | Having someone examine their mouth |  |  |  |  |  |
| 1805 | Having someone open their mouth |  |  |  |  |  |
| 1806 | Having a stranger touch them |  |  |  |  |  |
| 1807 | Having someone look at then |  |  |  |  |  |
| 1808 | The dentist drill |  |  |  |  |  |
| 1809 | The sight of the dentists drilling |  |  |  |  |  |
| 1810 | The noise of the dentist drilling |  |  |  |  |  |
| 1811 | Having someone put instruments in his/her mouth |  |  |  |  |  |
| 1812 | Choking |  |  |  |  |  |
| 1813 | Having to go to the hospital |  |  |  |  |  |
| 1814 | People in white uniforms |  |  |  |  |  |
| 1815 | Having the nurse clean their mouth |  |  |  |  |  |

**Section 19**

**Revised Child Manifest Anxiety Scale**

**To be filled by parents of children 6-7 years or those with poor communication skills**

**To be filled by children 8-12 years old**

Assess if your son/daughter would be afraid in the situations described below. Mark your answer with X.

|  |  | **Yes** | **No** |
| --- | --- | --- | --- |
| 1901 | I have trouble making up my mind |  |  |
| 1902 | I get nervous when things do not go the right way for me |  |  |
| 1903 | Others seem to do things easier than I can |  |  |
| 1904 | I like everyone I know |  |  |
| 1905 | Often I have trouble getting my breath |  |  |
| 1906 | I worry a lot of the time |  |  |
| 1907 | I am afraid of a lot of things |  |  |
| 1908 | I am always kind |  |  |
| 1909 | I get very upset easily |  |  |
| 1910 | I worry about what my parents will say to me |  |  |
| 1911 | I feel that others do not like the way I do things |  |  |
| 1912 | I always have good manners |  |  |
| 1913 | It is hard for me to get to sleep at night |  |  |
| 1914 | I worry about what other people think about me |  |  |
| 1915 | I feel alone even when there are people with me |  |  |
| 1916 | I am always good |  |  |
| 1917 | Often I feel sick in the stomach |  |  |
| 1918 | My feelings get hurt easily |  |  |
| 1919 | My hands feel sweaty |  |  |
| 1920 | I am always nice to everyone |  |  |
| 1921 | I am tired a lot |  |  |
| 1922 | I worry about what is going to happen |  |  |
| 1923 | Other children are happier than I am |  |  |
| 1924 | I tell the truth every single time |  |  |
| 1925 | I have bad dreams |  |  |
| 1926 | My feelings get hurt easily when I am fussed at |  |  |
| 1927 | I feel someone will tell me I do things the wrong way |  |  |
| 1928 | I never get angry |  |  |
| 1929 | I wake up scared some of the time |  |  |
| 1930 | I worry when I go to bed at night |  |  |
| 1931 | It is hard for me to keep my mind on my schoolwork |  |  |
| 1932 | I never say things that I shouldn’t |  |  |
| 1933 | I wriggle in my seat a lot |  |  |
| 1934 | I am nervous |  |  |
| 1935 | A lot of people are against me |  |  |
| 1936 | I never lie |  |  |
| 1937 | I often worry about something bad happening to me |  |  |

**Section 20**

**CFSS**

**To be filled by parents of children 6-7 years or those with poor communication skills**

**To be filled by children 8-12 years old**

Estimate the level of fear your son/daughter would perceive in the situations described below. Mark your answer with X.

How afraid is your son or daughter of …………

|  |  | **Not afraid at all** | **A little afraid** | **A fair amount** | **Pretty much afraid** | **Very afraid** |
| --- | --- | --- | --- | --- | --- | --- |
| 2001 | Having teeth out |  |  |  |  |  |
| 2002 | Injections |  |  |  |  |  |
| 2008 | The dentist drill |  |  |  |  |  |
| 2009 | Meeting the dentist |  |  |  |  |  |
| 2011 | Having someone look into his/her mouth |  |  |  |  |  |
| 2012 | Choking |  |  |  |  |  |
| 2013 | Having his/her teeth cleaned |  |  |  |  |  |
| 2014 | People in white uniforms |  |  |  |  |  |
| 2015 | Having to open his/her mouth wide |  |  |  |  |  |

**Section 21**

**Modified Child Dental Anxiety Scale (faces)**

**To be filled by parents of children 6- 7 years or those with poor communication skills**

**To be filled by children 8-12 years old**

Estimate the level of your anxiety in the situations described below. Mark your answer with X.

|  | **How do you feel about…** | Not worried | Fairly worried | Very worried |
| --- | --- | --- | --- | --- |
| 2101 | Having your teeth looked at |  |  |  |
| 2102 | Going to the dentist generally |  |  |  |
| 2103 | Having your teeth scraped and polished |  |  |  |
| 2104 | Having a mixture of gas and air which will help you feel comfortable for treatment but cannot put you to sleep |  |  |  |
| 2105 | Having a filling |  |  |  |
| 2106 | Being out to sleep to have treatment |  |  |  |
| 2107 | Having a tooth taken out |  |  |  |
| 2108 | Having an injection in the gum |  |  |  |

**Section 22**

**Corah’s Dental anxiety scale**

**To be filled by parents of children less than 8 years or those with poor communication skills**

**To be filled by children 8-12 years old**

**Mark your answer with x**

**2201. If your child had to go to the dentist tomorrow, how would (s)he feel about it. Mark the alternative that best describes the feelings**

………. (S)He will look forward to it as a reasonably enjoyable experience

………. (S)He wouldn’t care anyway or the other

………. (S)He would be a little uneasy about it

………. (S)He would be afraid that it would be unpleasant and painful

………. (S)He would be very frightened of what the dentist might do

**2202. When your child is in the waiting room in the dentist’s office for his/her turn in the chair, how would (s)he feel?**

………. Relax

………. A little uneasy

………. Tense

………. Anxious

………. So anxious (s)he may sometimes break out in sweat or almost feel physically sick

**2203. When your child is in the dentist’s chair waiting while he gets his drill ready to begin working on his/her teeth, how does (s)he feel?**

………. Relax

………. A little uneasy

………. Tense

………. Anxious

………. So anxious (s)he may sometimes break out in sweat or almost feel physically sick

**2204. Your child is in the dentist’s chair to have his/her teeth cleaned. While (s)he is waiting and the dentists is getting out the instruments which will be used to scrape his/her teeth around the gun, how does (s)he feel?**

………. Relax

………. A little uneasy

………. Tense

………. Anxious

………. So anxious (s)he may sometimes break out in sweat or almost feel physically sick

**THANK YOU VERY MUCH FOR YOUR TIME.**

**[NOTE: INTERVIEWERS EXPLAIN THAT SOMEONE WILL BE VISITING SUBSEQUENTLY AND PLEASE REMEMBER TO TURN TO THE NEXT PAGE.]**

**FINAL REMARKS ON THE QUESTONNAIRE**

**INTERVIEW CLOSING TIME………………………….DATE……………………………..**

##### LANGUAGE (s) THAT THE INTERVIEW WAS CONDUCTED IN………………….CODE…………

**INTERVIEWER’S COMMENTS……………………………………………………………………………..**

**……………………………………………………………………………………………………………………**

**…………………………………………………………………………………………………………………….**

**…………………………………………………………………………………………………………………….**

**SUPERVISOR’S COMMENTS……………………………………………………………………………..**

**……………………………………………………………………………………………………………………**

**…………………………………………………………………………………………………………………….**

**…………………………………………………………………………………………………………………….**

**Name……………………………………………. Signature/Date…………………………………………..**

**Information sheet for study participants**

**Digit sucking and oral health**

If you would like more information, have personal concerns, call  xxxx or send a mail to xxx The study is being undertaken by xxx, xxxx
